# Revisiting the Convective Like Boundary Layer Assumption in the Urban Option of AERMOD

**DOI:** 10.3390/atmos16121342

**Published:** 2025-11-27

**Authors:** Jonathan Retter, Robert Christopher Owen, Annamarie Leske, Michelle Snyder, Rhett Sargent, David Heist

**Affiliations:** 1 Oak Ridge Institute for Science and Education Research, Research Triangle Park, NC 27711, USA; 2 U.S. EPA Office of Research and Development, Center for Environmental Measurement and Modeling, Research Triangle Park, NC 27711, USA; 3 Johns Hopkins Applied Physics Labs, Laurel, MD 20723, USA; 4 WSP Global Inc., Durham, NC 27703, USA; 5 Department of Statistics, Virginia Tech University, Blacksburg, VA 24061, USA

**Keywords:** urban heat islands, remote sensing, urban sensible heat flux, dispersion modeling

## Abstract

Urban areas and their surroundings feature unique, horizontally inhomogeneous spatial distributions of land use and land cover, leading to urban heat islands (UHIs) for both air and land surface temperature that complicate the estimation of urban sensible heat flux. The urban dispersion option in AERMOD, the American Meteorological Society (AMS)/Environmental Protection Agency (EPA) Regulatory Model, incorporates this effect at night through a “convective like boundary layer” that modifies the single column meteorology based on a population number representative of the urban area. The model produces positive nighttime sensible heat flux values that often significantly overestimate observed values from the literature. This study re-examines the formulation of the AERMOD urban option assumptions, methodology, and original evaluation against a field study of a power plant in Indianapolis. We investigate replacing the population-based parameterizations of urban–surrounding temperature differences (ΔT) with observations of remotely sensed land surface temperature (LST) data from the Advanced Baseline Imager on the GOES-16/R/East geostationary satellite. We generated a monthly averaged, hourly, wind direction-dependent, clear sky land surface urban heat island ΔT database for 480 continental United States (CONUS) urban areas, as defined by the 2010 US Census. These ΔT values are used to advise city-specific horizontal advection corrections to sensible heat flux estimates that are neglected from simple energy balance models. The four cities of Cleveland, Amarillo, Atlanta, and Baltimore are highlighted, showing that the AERMOD predicted nighttime ΔT values are 794%, 416%, 1048%, and 758% higher, respectively, than the GOES-16 observations. These overestimated ΔT values in AERMOD lead to nighttime sensible heat flux values > 100 W/m^2^ that rival daytime values. However, using the GOES-16 observations as horizontal advection corrections to sensible heat flux results in trends that match the expected neutral to slightly positive nighttime values from observations recorded in the literature. The annual nighttime average in 2021 was −0.8 W/m^2^, 8.6 W/m^2^, 3.0 W/m^2^, and 3.1 W/m^2^ in Cleveland, Amarillo, Atlanta, and Baltimore, respectively, using this approach. Finally, reviewing the initial evaluation with the Indianapolis database against independent studies from the literature suggest that the AERMOD urban option inadvertently implements an urban heat island modeling approach to account for what was a low-level jet during the field study.

## 1. Introduction

Developed landscapes in urban areas alter the surface heat storage capacity from the surrounding natural unperturbed land. The increase in impervious surface and decrease in vegetated land cover tends to increase the Bowen ratio (sensible/latent heat flux ratio) while increasing the storage heat flux throughout the day. This complicates the interpretation of the urban sensible heat flux and, in turn, convective atmospheric turbulence that is key for dispersion modeling. Urban infrastructure can act as a “battery”, storing more heat from the sun than surrounding vegetated areas during the day and releasing it back to the atmosphere in the evening and night, increasing the air temperature and causing the well-known UHI [1]. This additional heat release, along with physical constraints from structures that contribute to mechanical turbulence in urban areas, modifies the local meteorological conditions which are used as inputs for dispersion models. These unique meteorological conditions manifest themselves as “urban options” in dispersion models such as AERMOD [2], the focal point of this work. As a Gaussian dispersion code, AERMOD relies on parameterizations to compute concentrations, which struggle to capture the spatial and temporal variations in urban environments. Given that 80% of the population of the United States lives in urban areas [3], it is key to understand the effects of this heat release on the atmospheric stability and turbulence for accurate dispersion predictions.

A diurnal sensible heat flux curve for non-urban environments remains in phase with solar radiation and is positive during the day (heat to the atmosphere) yet negative at night (heat to the surface). However, in urban areas, the diurnal trend is modified, with larger magnitudes [4,5] reaching maximum values later in the afternoon, and potentially remaining positive after sunset and throughout the night [6,7]. The latter suggests convective conditions (or unstable Monin–Obukhov boundary layer classification) exist even at night in urban environments, as several authors have noted with independent observations in London [8], Beijing [9], Rome [10], and Basel [11].

However, the local details of the land cover complicate the analysis from discrete sensors. Eddy covariance measurements for different land cover types from the Basel Urban Boundary Layer Experiment (BUBBLE) [7] in Basel, Switzerland, the Joint Urban 2003 field experiment in Oklahoma City, United States [12], and the U.S. Environmental Protection Agency’s Regional Air Pollution Study in St. Louis, United States [13] within the same urban/surrounding areas demonstrate significant variations for nominally the same net radiation conditions for each city. Each study illustrates the importance of surface storage flux and advection due to horizontally inhomogeneous land cover. For the BUBBLE study, six stations (three urban, one suburban, two rural) over one summer month in 2002 were studied by Christen et al. [7], where all urban sites featured sensible heat flux away from the surface throughout the night, near zero nocturnal sensible heat flux for the suburban site, and sensible heat flux in phase with the solar radiation for the rural site (negative at night).

Hanna et al. [12] analyzed 10 discrete sites through suburban/urban Oklahoma City and offered general conclusions to guide the development of dispersion models. Recommendations regarding sensible heat flux included a nocturnal value of +10 W/m^2^ in downtown areas, albeit with stability class complications encouraging the use of a minimum Obukhov length [14], and a positive time shift in the diurnal solar radiation curve. For storage heat flux, the ratio with respect to the net radiation in suburban areas was 0.05–0.1 for day and night, whereas downtown urban areas during the day was 0.15–0.5 and increasing at night to 0.2–2 depending on the specific site.

Finally, Ching et al. [13] compared the advection of heat flux due to UHI or land cover at city and local scales in St. Louis with translating aircraft measurements of turbulent sensible heat flux over the city center and three discrete sensor towers in an industrial/commercial area, a residential area, and an agricultural rural area. Aircraft measurements suggested the presence of cold air advection upwind of the urban heat island due to a skew in the spatial profile through the city center. Single-site observations reached similar conclusions of Christen et al. and Hanna et al.—urban sensible heat flux remains slightly positive at night and diurnal curves are out of phase with the net radiation, although ground cover differences matter. These complexities in determining the urban sensible heat flux have led to a documented need for increased/routine monitoring networks in urban areas to understand these effects [15–17].

The motivating source for this work was to compare urban heat flux modeling in the AERMOD modeling system [2] to observed values from the literature. This exact goal was included in the Hanna et al. [12] study discussed above, where the authors state “it would be useful to check the recommended flux ratios with the various urban meteorological pre-processor and/or energy flux software programs available [such as… the AERMOD method…]”. We do that here, beginning with a brief overview of the convective portion of the urban option in AERMOD, a proposed remotely sensed observation-based approach to provide city-specific advection corrections, and the application of these advection corrections to AERMOD. This approach is compared to the Indianapolis Urban Power Plant Study used as the original evaluation of the AERMOD urban option.

## 2. Theory

### 2.1. Urban Energy Balance

The full volume-based urban energy balance can be written as [18]

(1) $$Q^{*}+Q_{F}=Q_{H}+Q_{E}+\Delta Q_{s}+\Delta Q_{A}$$

where $Q^{*}$ is the net radiation, $Q_{F}$ is the anthropogenic heat, $Q_{H}$ is the turbulent sensible heat flux, $Q_{E}$ is the latent heat flux, $\Delta Q_{s}$ is the storage of sensible heat, and $\Delta Q_{A}$ is the advection of heat. From a dispersion perspective, the components of interest are the sensible, advective, and anthropogenic heat sources, as they modify the turbulence profile through a convective velocity scale. Anthropogenic sources are difficult to determine and are usually left as the residual component after observations of all others; therefore, we limit our analysis to applying horizontal advection corrections using LST data.

### 2.2. Horizontal Advection

We follow the work of other authors considering horizontal advection of sensible heat flux, beginning with a brief derivation of the simplified expression used in this work. The differential relation for heat conservation is given by Stull [19] (Stull’s Equation (3.4.5b)).

(2) $$\overset{‾}{\rho }C_{P}\left[\frac{\partial \overset{‾}{\theta }}{\partial t}+\frac{\partial \bar{u_{j}^{\prime }\theta^{\prime }}}{\partial x_{j}}+\bar{u_{j}}\frac{\partial \overset{‾}{\theta }}{\partial x_{j}}+\frac{v\partial^{2}\overset{‾}{\theta }}{\partial x_{j}^{2}}\right]+\frac{\partial Q^{*}}{\partial x_{j}}+Q_{E}=0,$$

where $\overset{‾}{\theta }$ is the potential temperature, $x_{j}$ and $u_{j}$ are the spatial and velocity “jth” coordinate, respectively, and v is the diffusivity of heat. The first term is the storage of heat, followed by the divergence of turbulent heat flux, the advection of heat, the molecular conduction of heat, the radiation divergence, and finally the source term associated with latent heat. Assuming molecular conduction is small, neglecting additional heat source terms (latent heat in this case), and considering a flow direction aligned in u with the vertical component in w, results in the expression from Pigeon et al. (Equation (2) in [20]) of

(3) $$\overset{‾}{\rho }C_{P}\left[\frac{\partial \overset{‾}{\theta }}{\partial t}+\frac{\partial \bar{w^{\prime }\theta^{\prime }}}{\partial z}+\overset{‾}{u}\frac{\partial \overset{‾}{\theta }}{\partial x}+\overset{‾}{w}\frac{\partial \overset{‾}{\theta }}{\partial z}\right]+\frac{\partial Q^{*}}{\partial z}=0.$$

Looking only at the horizontal advection term, $\overset{‾}{u}\frac{\partial \overset{‾}{\theta }}{\partial x}$, we integrate vertically to a height, h, to reach the advective portion of the expression from Ching et al. [13] (see Equation (7)) and Hidalgo et al. [21] (Section 4.1 of [21]).


(4) $$\Delta Q_{A}=\overset{‾}{\rho }C_{p}\int_{0}^{h}\overset{‾}{u}\frac{\partial \overset{‾}{\theta }}{\partial x}\text{dz}.$$

Approximating this integral relation over a vertical distance Δz and estimating potential temperature as actual temperature, we reach the reduced expression of Sproken-Smith et al. [22] (see Equation (2.1)) and Cuxart et al. [23] (see Equation (3)) of

(5) $$\Delta Q_{A}=\rho C_{p}u\frac{\Delta T}{\Delta x}\Delta z.$$

ΔT is the difference in temperature related to the spatial scales considered (Δx,Δz), which we consider the difference between the urban and surrounding areas.

The magnitude of horizontal advection of sensible heat flux is inherently related to the scale considered and the details of the land cover class. Pigeon et al. [20] analyzed flux observations from the coastal city of Marseille, France as a function of distance from the shore, showing significant horizontal advection contributions from sea breeze near 200 W/m^2^ at the shoreline that decayed to 20–80 W/m^2^ > 2 km inshore. An analysis of a scintillometer study in London by Crawford et al. [24] illustrated the effect of wind direction on local scale Δ*Q*_*A*_, with estimates that varied from ±134.4 W/m^2^ with winds from the southwest to ±20.8 W/m^2^ with winds from the east. However, horizontal advection may not always be a significant source of sensible heat. Mesoscale advection was estimated as less than ±30 W/m^2^ by Sproken-Smith et al. [22] in suburban areas of Christchurch, New Zealand during winter. Hidalgo et al. [21] estimated horizontal advection from an urban breeze study in Toulouse, France and deemed it negligible at only 6 W/m^2^. However, such small values may be relevant for very detailed studies such as glacier melt rate [25] or may be critical to consider at night when estimates of sensible heat flux from energy balance models are small, where ~10 W/m^2^ adjustments could theoretically alter the stability class of the model.

### 2.3. AERMOD Urban Option

**Figure 1.**
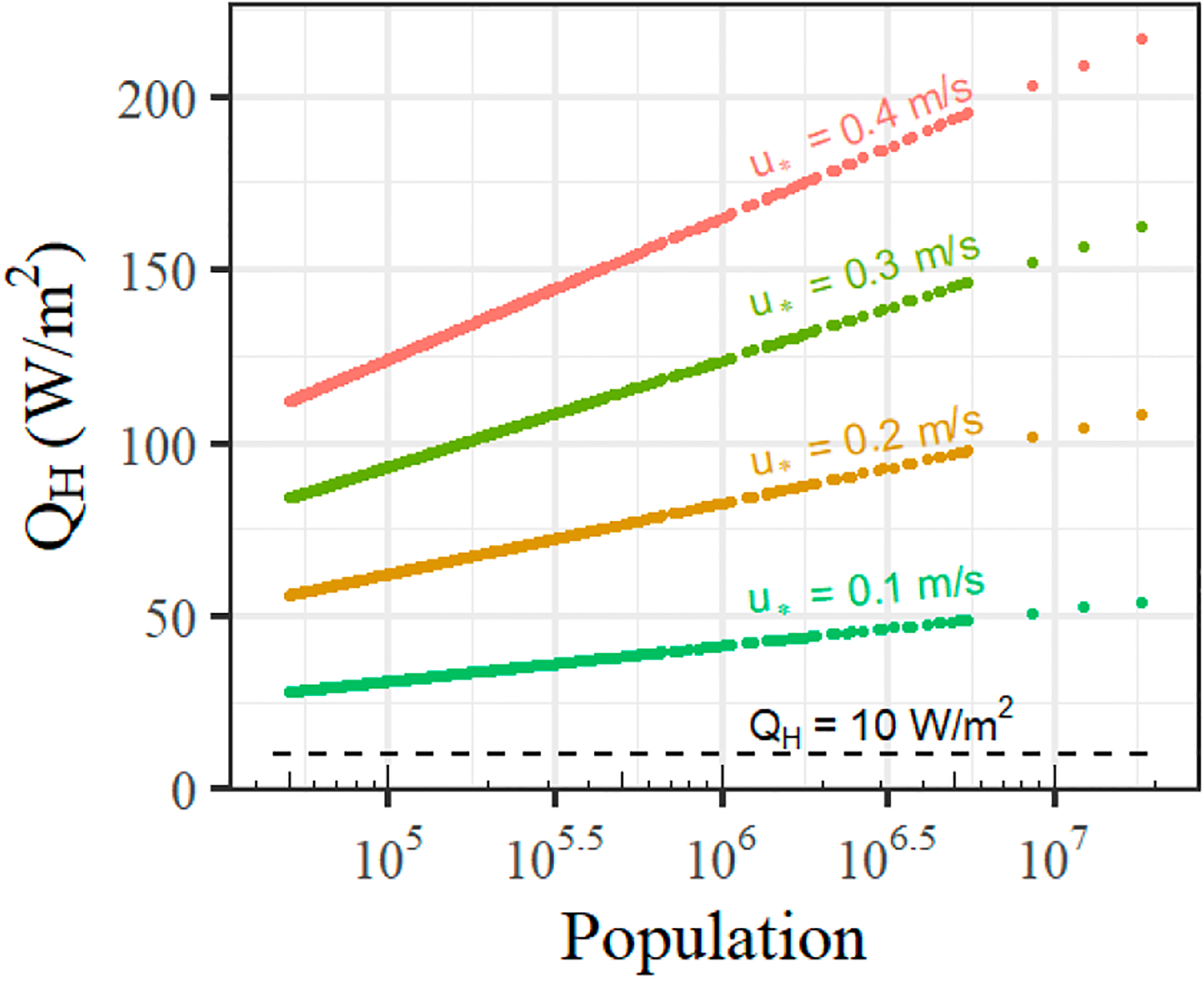
Existing AERMOD nocturnal sensible heat flux values compared to constant value of 10 W/m^2^ from Hanna et al. [12].

The current formulation of the energy balance in AERMOD (v22112) for rural and daytime urban areas does not include advective or anthropogenic sources of sensible heat flux and simplifies the energy balance, stating the ground heat flux is 10% of the radiation throughout the day, leading to a simple expression of sensible heat as [26]

(6) $$Q_{H}=\frac{0.9Q^{*}}{1+1/B_{o}},$$

where $B_{o}$ is the Bowen ratio. This formulation ensures that the sensible heat flux always matches the sign of the solar radiation. $Q_{H}$ is directly replaced at night through a procedure parameterized by a single population input, P, which guides the nighttime canopy layer UHI ΔT modeling through an empirical expression [27] as

(7) $$\Delta T=12.0\left[0.1\text{ln}\;\frac{P}{P_{o}}+1.0\right],$$

where $P_{o}$ is a reference population of 2 million. This horizontal ΔT is applied directly to the calculation of the vertical sensible heat flux as

(8) $$Q_{H}=\alpha \rho C_{p}\Delta Tu_{*},$$

where α is a nondimensional bulk heat transfer coefficient set equal to 0.03, and $u_{*}$ is the friction velocity (m/s). Given the horizontal nature of ΔT, this expression parallels the practical advection relation of Equation (5), where the friction velocity replaces a reference velocity, and the bulk heat transfer coefficient assumes the role of $\frac{\Delta z}{\Delta x}$. The result from Equation (8) replaces the heat flux calculated from Equation (6), and forces a positive, sizeable sensible heat flux value for all hours at night. For reasonable inputs ($\rho =1.225\;\text{kg}/{\text{m}}^{3},\;C_{p}=1004\;\text{J}/\text{kg}-\text{K},\;u_{*}=0.2\;\text{m}/\text{s},\;\Delta T_{\text{air}}\sim 10\;\text{K}$ at P=400,000), the modeled sensible heat flux is quite large at ~74 W/m^2^ and quickly becomes unreasonable and rivals or surpasses daytime flux estimates for higher values of $u_{*}$. Considering the populations of the largest 480 urban areas from the 2010 US Census, Figure 1 displays the predicted nocturnal sensible heat flux over a range of relevant $u_{*}$ against the suggested constant value from Hanna et al. [12].

Additional modifications are made to the mixing height and mechanical turbulence through the friction velocity as a function of this modified heat flux, as described in the AERMOD formulation document [26]. However, given the erroneous heat flux values used as inputs, these are viewed as a propagation of error to be revisited in future work. There are many ways to remedy this overestimation in AERMOD, such as simply changing the value of α in Equation (8) or adding this inherently horizontal advection component to the existing value computed from AERMET in Equation (6), the meteorological precursor to AERMOD, rather than a direct replacement, but we choose to focus on ΔT observations that are more representative of the sensible heat flux than population numbers.

### 2.4. Proposed AERMOD Urban Energy Balance

The existing formulation based on population is always positive and independent of local climate zones, seasons, time of day (albeit $u_{*}$ does change throughout the day), and directional ground cover, all of which are expected to alter the sensible heat flux. In this work, we estimate city-specific corrections to the existing energy balance model by adding city-scale horizontal advection using directional fetch-dependent remotely sensed ΔT observations. A similar directional procedure is already used in AERMET to estimate the surface roughness, albedo, and Bowen ratio from national land cover data [28]. Note that we are not proposing to replace the existing AERMOD air ΔT values with the remotely sensed land surface ΔT values, but rather replacing the erroneous formulation of using horizontal temperature differences directly as vertical sensible heat flux. Horizontal differences must be considered as city-specific advection corrections.

First, however, the sensible heat flux expression in AERMET (Equation (6)) was designed for a rural reference and requires slight modification for urban use. For each nighttime hour, $\Delta Q_{s}/Q^{*}$ is adjusted to balance the net radiation ($\Delta Q_{s}/Q^{*}\sim 1$), to allow $Q_{H}=0$, or neutral, at night before the advective additions are made. This is consistent with (1) suggestions in Luhar et al. [4] of making the urban boundary layer neutral when rural stability is stable, (2) the recommendations of Hanna et al. [12] of $\Delta Q_{s}/Q^{*}$ increasing at night to 0.2–2 as discussed previously, and (3) the 20-day average observation of $\Delta Q_{s}/Q^{*}>1$ at night seen during BUBBLE [29]. Written explicitly, the proposed contributions to convective turbulence in urban areas are

(9) $$Q_{H}+\Delta Q_{A}=\left\{\begin{array}{rr} \frac{0.9Q^{*}}{1+1/B_{o}}+\rho C_{p}u\frac{\Delta T}{\Delta x}\Delta z, & \text{if}\;\text{daytime}\\ 0+\rho C_{p}u\frac{\Delta T}{\Delta x}\Delta z, & \text{if}\;\text{nighttime} \end{array}.\right.$$

**Figure 2.**
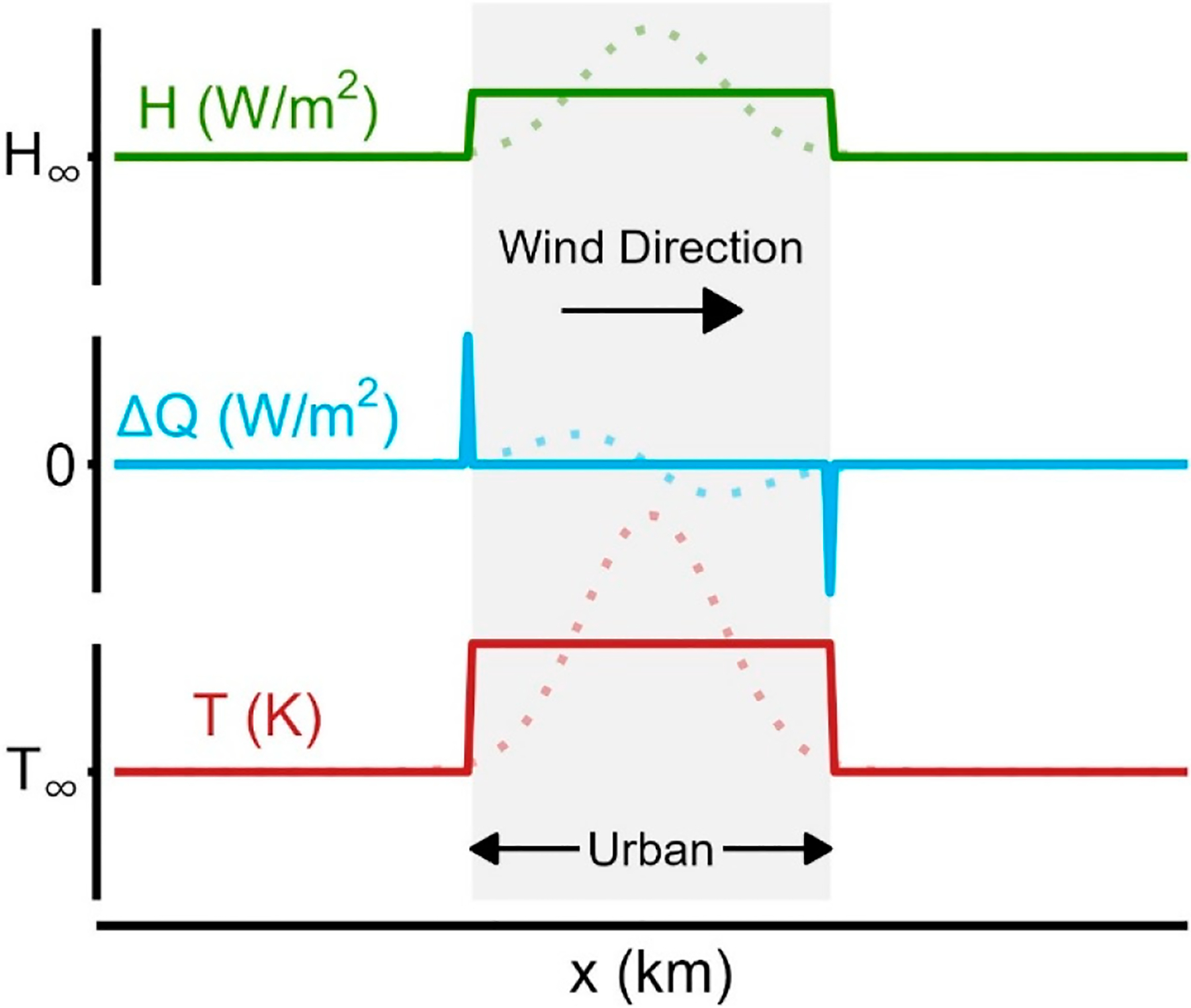
Idealized step change in temperature from a reference value of $T_{\infty }$ (**bottom**) as horizontal advection of heat flux (**middle**) resulting in a net change in heat flux from a reference value of $H_{\infty }$ (**top**). Solid curves represent the simplification pursued in this work while faded dots represent a more realistic temperature change at higher spatial resolution.

We consider ΔT as a step change in temperature [30,31] related directly to sensible heat flux [32], where ΔT is applied at the interface of the urban–surrounding area. A motivating illustration is shown as the solid curves in Figure 2, with a theoretical spatial step change in temperature for an urban area shown in red. Considering a wind direction from left to right and applying Equation (9) results in a $+\Delta Q_{A}$ at the upstream interface coupled with a $-\Delta Q_{A}$ at the downstream interface. Taking the urban area as one “unit”, a crude approximation, this acts as a total sensible heat flux increase for the urban area. With inspiration from Ching et al. [13] (see Figure 4 within [13]), more representative curves are shown as faded points with increased spatial resolution of each respective curve.

## 3. Materials and Methods

**Figure 3.**
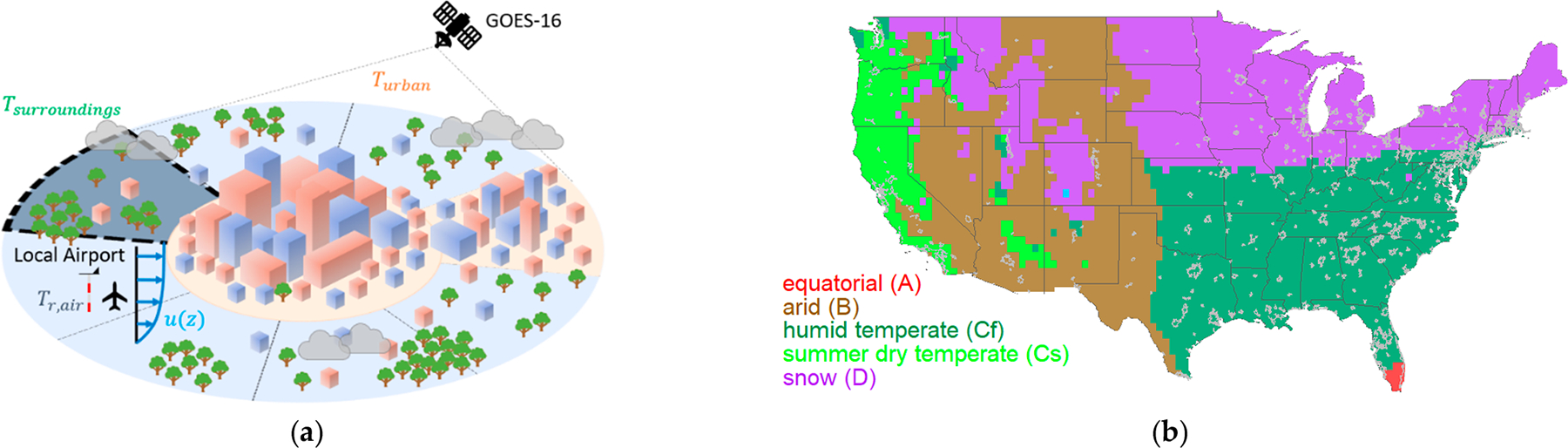
(**a**) Illustration of the objective of this study, considering the upwind fetch. (**b**) Köppen–Geiger climate zone groups and locations of the 480 US urban areas of interest.

Considering advection corrections across the United States requires a data source that can cover the entire spatial domain. Therefore, we chose to estimate these advection corrections using land surface temperatures from the GOES-16 Advanced Baseline Imager (ABI) applied to administrative urban boundaries and a surrounding buffer area. The databases for LST, urban boundaries, and land cover class are discussed in this section. A directional approach is implemented to best account for the change in land cover surrounding urban areas for different wind directions. As illustrated in Figure 3a, based on the local wind direction, we consider the upwind LST value as the “surrounding” temperature. This is true even if the fetch includes another urban area, such as the illustrative case of Figure 3a if flow were from the right to left. Land cover for each fetch segment and urban area along with their respective climate zones are analyzed in the Supplemental Material to compare against the surface temperature towards developing general guidance for CONUS cities.

### 3.1. Urban Description

#### 3.1.1. Census Boundaries

US Census definitions of urban areas [33], or population centers of over 50,000 people, were used explicitly as administrative boundaries. Data from 2010 was used, as 2020 Census data was unavailable at the start of this study, resulting in 480 urban areas within the CONUS. Shape files are available for free from the US Census Department and are shown in gray over major Köppen–Geiger climate zone groups in Figure 3b. Temperate climate zones were split into humid (Cf) and summer dry (Cs) temperate zones to isolate trends on the west and east coast of CONUS. There are 2 cities in equatorial, 49 in arid, 234 in humid temperate, 68 in summer dry temperate, and 127 in snow climates.

#### 3.1.2. National Land Cover and Fetch Considerations

Land cover information is provided from the United States Geological Survey from 2019. The landcover data was down sampled from 30 m × 30 m resolution to the resolution of the GOES-16 LST data (2 km × 2 km) and summarized for all valid pixels within each US Census-defined urban area and corresponding surrounding sectors. The ~66.7× difference in spatial scales complicates defining a single land cover class for each GOES-16 pixel, as many land cover classes combine to create a single GOES-16 pixel. Using the local climate zone (LCZ) classification [34,35] was considered, given the increased number of urban classifications from 3 to 10 and usefulness in the direct connections to land surface temperature [36]. However, AERMOD currently utilizes the National Land Cover Database (NLCD) to define the surface albedo, surface roughness, and Bowen ratio; therefore, to maintain consistency with the code of interest, the 2019 version was also used here.

**Figure 4.**
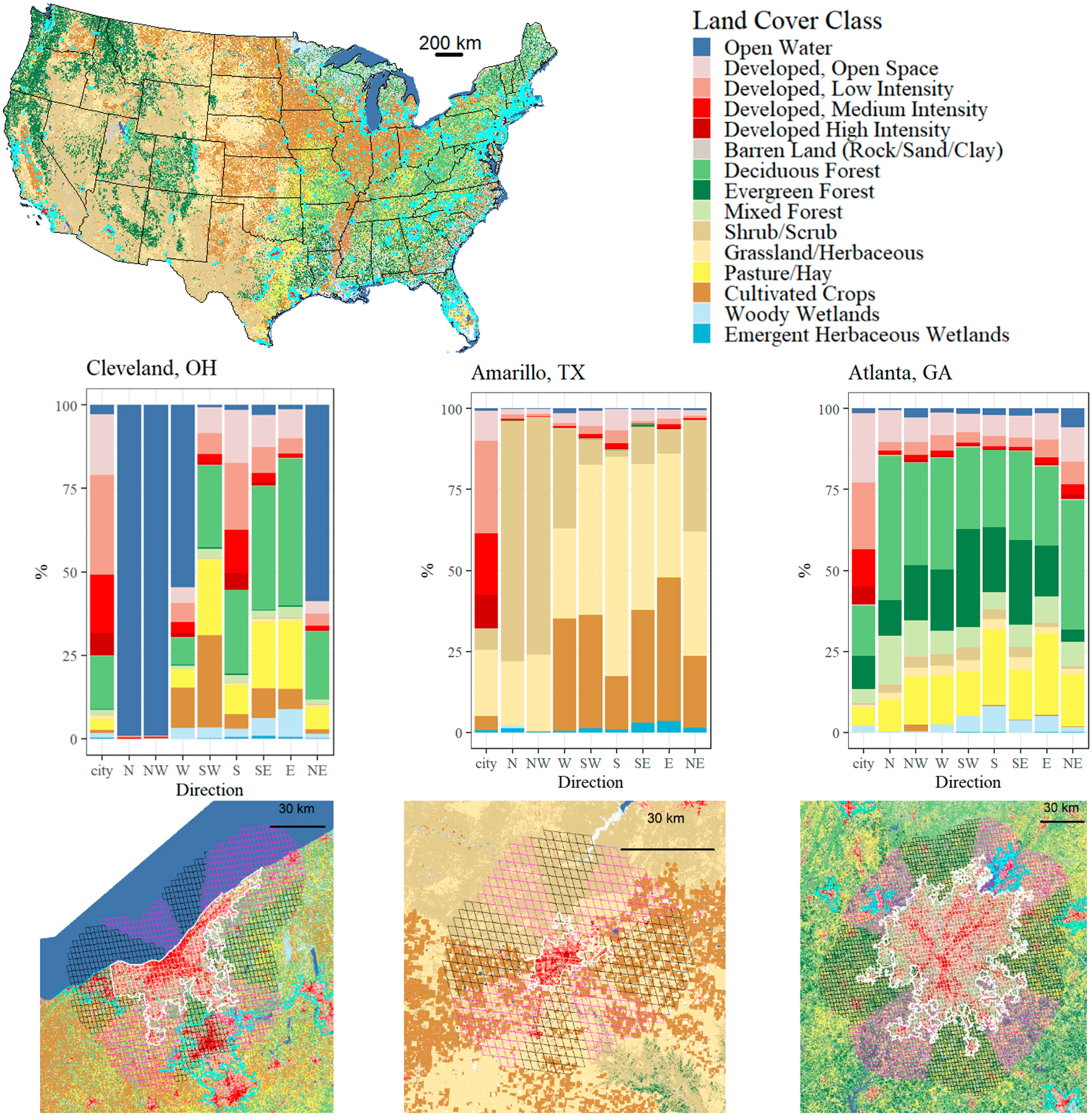
Example overlay of the National Land Cover Database and the GOES surrounding grid points. Example cities for each climate zone (**left** = Cleveland, Ohio; **middle** = Amarillo, Texas; **right** = Atlanta, Georgia).

The change in temperature experienced by the boundary layer is a function of the direction in which it passes over the urban area. The structure of the velocity and turbulence profile will adapt based on what the flow “sees” below/in front of it in the form of surface and thermal roughness. Taking the centroid of each US Census-defined city, we generate 45-degree sectors corresponding to the eight major directions and use a 30 km buffer from the urban area boundary to define surrounding sectors. Note that this constant buffer size may be roughly equal to the urban area, larger than it, or smaller than it, as illustrated in Figure 4 with examples of Cleveland, Amarillo, and Atlanta.

Figure 4 depicts the NLCD for CONUS with an overlay of the US Census-defined urban areas and highlights three cities in separate climate zones—Cleveland, OH in the snow climate, Amarillo, TX in the arid climate, and Atlanta, GA in the humid temperate climate. The bottom row of the figure illustrates the resolution of the GOES-16 LST grid on the NLCD data for each urban area highlighted in white. Other nearby urban areas are outlined in blue. The directional sectors are color-coded, with N-S-E-W in black and all angled sectors in purple. The center row of Figure 4 summarizes the land cover for each sector. For example, Cleveland has significant directional dependence, where flow from the north is from open water compared to flow from the south through another urban area (Akron, OH, USA). Surface ΔT results corresponding to these cities are shown in the Supplemental Material.

### 3.2. Temperature Measurements: GOES-16 Advanced Baseline Imager

The Advanced Baseline Imager (ABI) from the NOAA/NASA GOES-16/R/East geostationary satellite was used for hourly measurements of both LST and sea surface temperature (SST) for the CONUS at an angular resolution of 56μradians, relating to ~2–4 km spatial resolution over CONUS. Note that with GOES-16 centered at 75.2 degrees west, the spatial resolution decreases from the east coast to west coast. Valid SST observations are added to the 2500 × 1500 LST grid for each hour outside of the CONUS boundary. Observations are limited to clear sky conditions, suggesting that any surface urban heat island measurements are biased high as clear sky conditions optimize the solar radiation to the surface. Effects of the average wind speed during each measurement were not considered in this work. A monthly averaged, hourly land and sea surface temperature product was developed to improve the success rate of having valid data in an area. Data is freely available via the NOAA Comprehensive Large Array-data Stewardship System (CLASS) website or Amazon Web Services (AWS) with interfaces in R or Python. Full details on the temperature algorithms for LST [37] and SST [38] are available from NOAA, with brief descriptions included below.

The GOES-16 LST is calculated from 11.19μm and 12.27μm wavelengths after correcting for surface emissivity and atmospheric absorption [37]. Instrument exposure times were ~2 min 37 s for LST, typically at the beginning of the hour. We analyzed LST data from 2018 to 2022 to identify step changes in the surface temperature entering each city in CONUS, although only data from 2021 with a corresponding valid SST file is shown in this work for brevity. SST is calculated from three wavelengths of 3.9μm,11.19μm, and 12.27μm integrated over 1 h.

**Figure 5.**
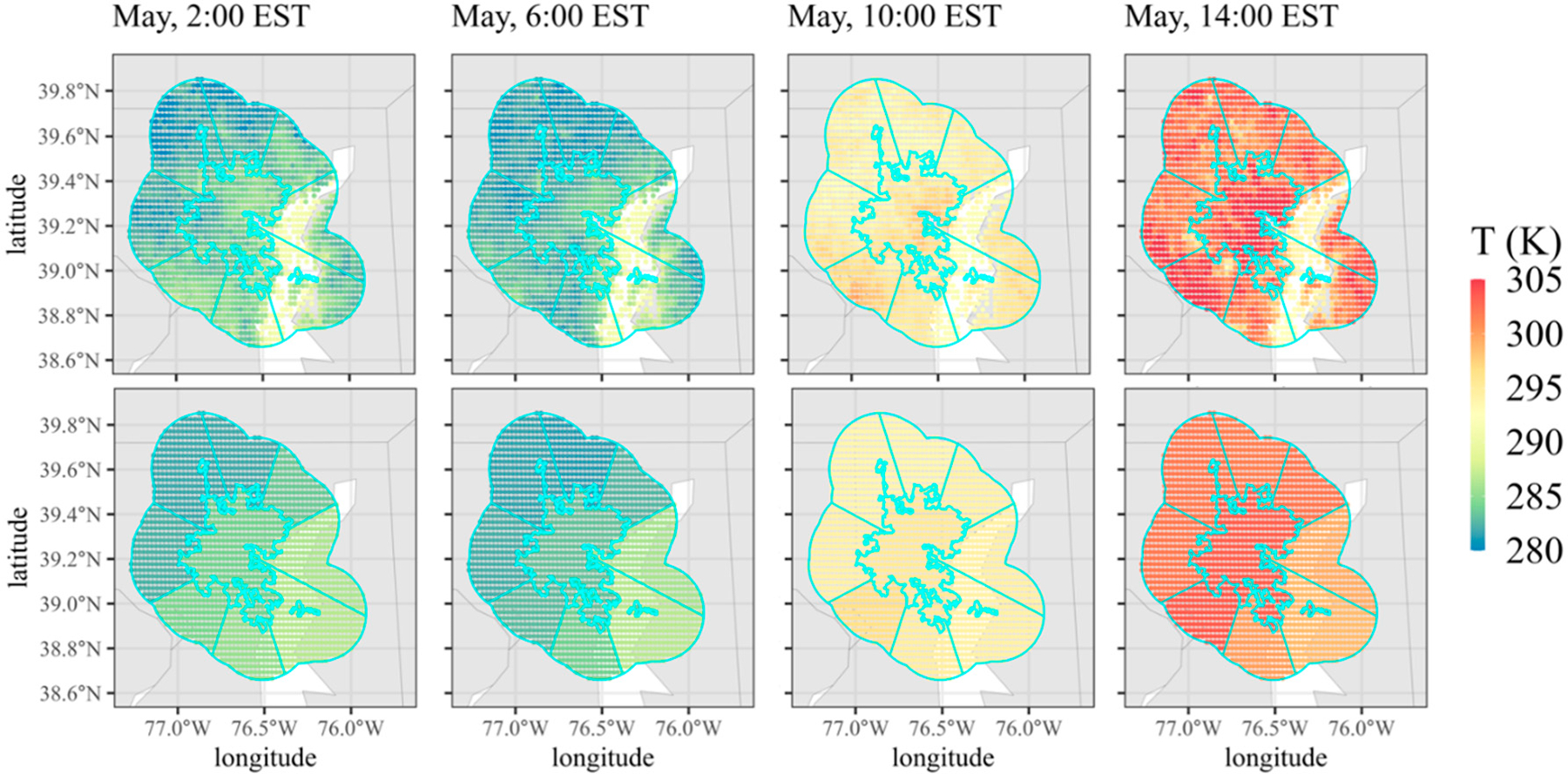
Time-averaged LST and SST values for Baltimore, MD in May 2021 for 2:00, 6:00, 10:00, and 12:00 EST (**top row**) and the corresponding spatial average for each sector (**bottom row**).

Coupling the resolution of the GOES ABI with the urban–surroundings definitions used in this work, the average number of pixels in the urban area was ~72 with an average rural sector size of ~89 pixels. Figure 5 presents representative data for Baltimore, Maryland, where 305 GOES pixels define the urban area with an average of 176 pixels for each surrounding sector. For each month, hourly average combinations of LST and SST are produced for each urban area and the surrounding directional rural sectors, as shown in the first row of Figure 5. To simplify potential inputs for dispersion application, we average all temperatures within each section to produce a single ΔT for each direction (bottom row of Figure 5). We experimented with changing the rural buffer size from 10, 20, 30, and 40 km to understand the effect on ΔT. While the effects were city-specific, the average of all ΔT values combined varied only by 0.03 K for each rural buffer size over all 480 cities. A larger effect of an average increase of 0.09 K was observed by considering urban areas smaller than the Census definition.

### 3.3. Indianapolis Urban Power Plant Study

A field study in Indianapolis from September to October in 1985 measured ground level concentrations of sulfur hexafluoride (SF6) from an 83.8 m stack near the city center to develop a database to model urban dispersion. The study was summarized by Murray et al. [39], where observations were compared to the EPA’s RAM model and the Industrial Source Complex model using urban “modes”. These models were precursors to AERMOD, the EPA’s current preferred Gaussian dispersion model and the focus of this work. This Indianapolis dataset was also used to evaluate the AERMOD urban option and was used “to some extent in the model development” [40]. Given this was the only urban database used in the validation of the AERMOD urban option, we revisit the comparison here to highlight how the existing formulation is not physically representative of the field study used to evaluate it.

We obtained the Indianapolis data from the Modelers Data Archive [41]. In addition to concentration observations for 177 monitors in arcs from 250 m to 12 km downwind of the source, it contains meteorological information for an urban, suburban, and rural locations near the source including turbulent kinematic heat flux. Additional, but more limited meteorological information, is available on the bank tower and nearby National Weather Service (NWS) site at the Indianapolis Airport. The original AERMOD validation utilized the NWS site for the surface meteorology, which is the farthest site from the source (see the latitude/longitude of 39.733° N86.267° W in the INDSURF.RUR file in the Indianapolis database files available on the EPA Support Center for Regulatory Atmospheric Modeling (SCRAM) [42]). Additional analysis of this study relevant to this work was performed by Hanna and Chang [14,43] and is discussed along with our work in Section 4.2.

## 4. Results

The sector-based averaging procedures using LST and SST, as described in Section 3, are performed for 480 urban areas in CONUS. Individual temperature differences for each city are tabulated in input files for potential future use and comparisons are made for climate zone and ground cover dependence as seen in Supplemental Material to this document. They are applied as horizontal advection corrections to a modified energy balance system. This is offered as a potential replacement for the existing AERMOD urban energy balance. Four illustrative example cities are discussed here, corresponding with the land cover discussion and local advection scale case study in Section 5.1 with select coastal cities examined in the Supplemental Material. The Supplemental Material also includes seasonal diurnal LST values according to the climate zone or land cover class [44–46]. These values can be used for cities or towns not included in the urban database.

### 4.1. Horizontal Advection Correction to Energy Balance

The climate zone and land cover profiles can provide city-generic guidance on ΔT values but mitigate any unique trends from individual cities. Therefore, we apply the city-specific directional ΔT values to surface meteorological input files from AERMET, the meteorological pre-processor for AERMOD. Surface files containing hourly data from 2021 for a variety of US cities are available from the EPA’s Human Exposure Model (HEM) website [47]. These AERMET runs were generated in a manner similar to most regulatory applications, i.e., with 2 min automated surface/weather observing systems data from major airports, including the adjust $u_{*}$ option.

**Figure 6.**
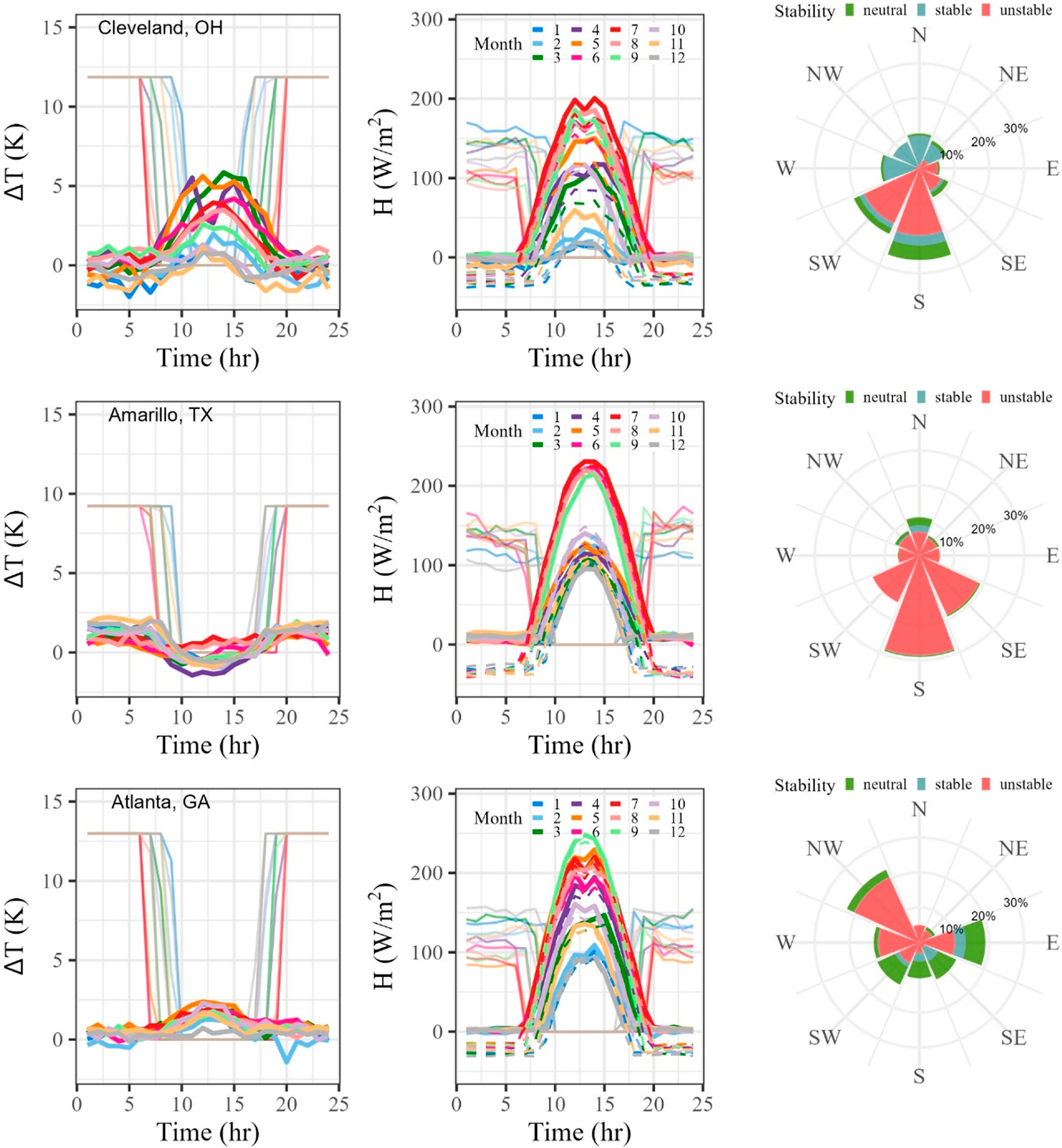

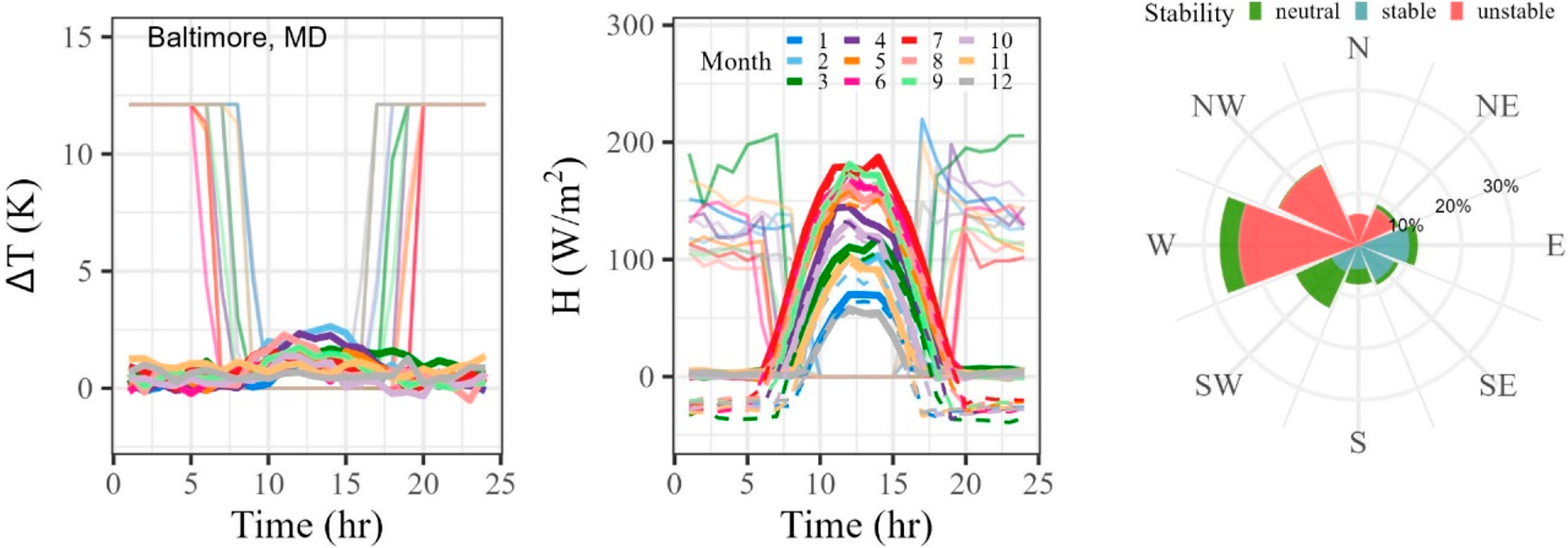
Example ΔT values for the LST approach in bold lines and the population parameterization in thin lines (**left**). Sensible heat flux profiles for the rural AERMET outputs in dashed lines, the AERMOD urban option based on population in thin lines, and the LST approach in bold lines (**middle**). Directional nocturnal stability plots based on wind direction (**right**). Each row is a new city, Cleveland, Amarillo, Atlanta, and Baltimore.

We use Equation (9) to add the contribution of the advective component to this now neutral baseline at night and to the existing convective conditions during the day for every hour of the year, selecting the wind direction dependent ΔT for each hour. For the spatial variables in Equation (9), Δx is 2 km (the spatial resolution of the GOES-16 LST product) and Δz is 2 m (typical height of NWS station where temperature is recorded). Both Sproken-Smith et al. [22] and Cuxart et al. [23], the motivating works for Equation (5), utilized the height of the air sensors as the vertical distance. This allows us to adjust the city-generic heat flux curve by city-specific directional inputs. Figure 6 provides a summary of these efforts for the four representative cities shown previously in Figure 4 and again in the Supplemental Material (Figure S1) such that the reader can compare directly against the directional ground cover and the raw directional data, respectively. Additional analysis focused on major coastal cities is included in Section 1.4 of the Supplemental Material to highlight the importance of nearby large bodies of water.

The first column of Figure 6 shows the hourly average ΔT weighted by the wind direction for each hour and corresponding to the month for our method, Equations (2) and (3), and the current urban option in AERMOD. This population expression only applies at night, resulting in a constant ΔT that falls to zero during the day. See Appendix A for a discussion of the issues with a constant ΔT. Considering the maximum of the nighttime nocturnal ΔT values for each city, the AERMOD predicted values are 794%, 416%, 1048%, and 758% higher than the observations for Cleveland, Amarillo, Atlanta, and Baltimore, respectively.

Diurnal sensible heat flux curves are shown in the second column of Figure 6. Note that the modifications proposed in this work result in heat flux values near zero at night, whereas the existing formulation produces unphysical values that are near or above daytime values. Considering an average over all months, Equation (9) gives a sensible heat flux of −0.8 W/m^2^, 8.6 W/m^2^, 3.0 W/m^2^, and 3.1 W/m^2^ for nighttime hours in Cleveland, Amarillo, Atlanta, and Baltimore, respectively, with corresponding average AERMOD predictions of 120.8 W/m^2^, 127.1 W/m^2^, 115.0 W/m^2^, and 126 W/m^2^.

Finally, the nocturnal stability class corresponding to each wind direction is shown in the third column of Figure 6. Neutral conditions were assumed when the absolute value of the sensible heat flux was less than 1 W/m^2^. Occurrences are presented as the percentage observed during the entire year of 2021. The resulting stability classes tend to match the literature findings. It is not uncommon to find the stability of an urban area as unstable at night. Rather it occurs most of the time (see [11], Figure 16.6), suggesting the use of convective dispersion coefficients. However, Hanna and Chang encourage the use of a minimum Obukhov length [14] to couple the positive heat flux with stable dispersion coefficients. AERMOD currently incorporates a variation in this “minimum L” value suggestion of Hanna and Chang by taking the absolute value of the urban-calculated Obukhov length to force a stable boundary layer in conjunction with positive nighttime sensible heat flux values. For the four urban areas shown in Figure 6, unstable nocturnal stability is found 53%, 92%, 62%, and 55% of the time for Cleveland, Amarillo, Atlanta, and Baltimore, respectively, with representative heat flux values of −10 to 20 W/m^2^ like those seen in the literature. Further work is required to see the effect of these stability classes coupled with reduced heat flux on concentration predictions.

### 4.2. Application to the Indianapolis Urban Power Plant Study

**Figure 7.**
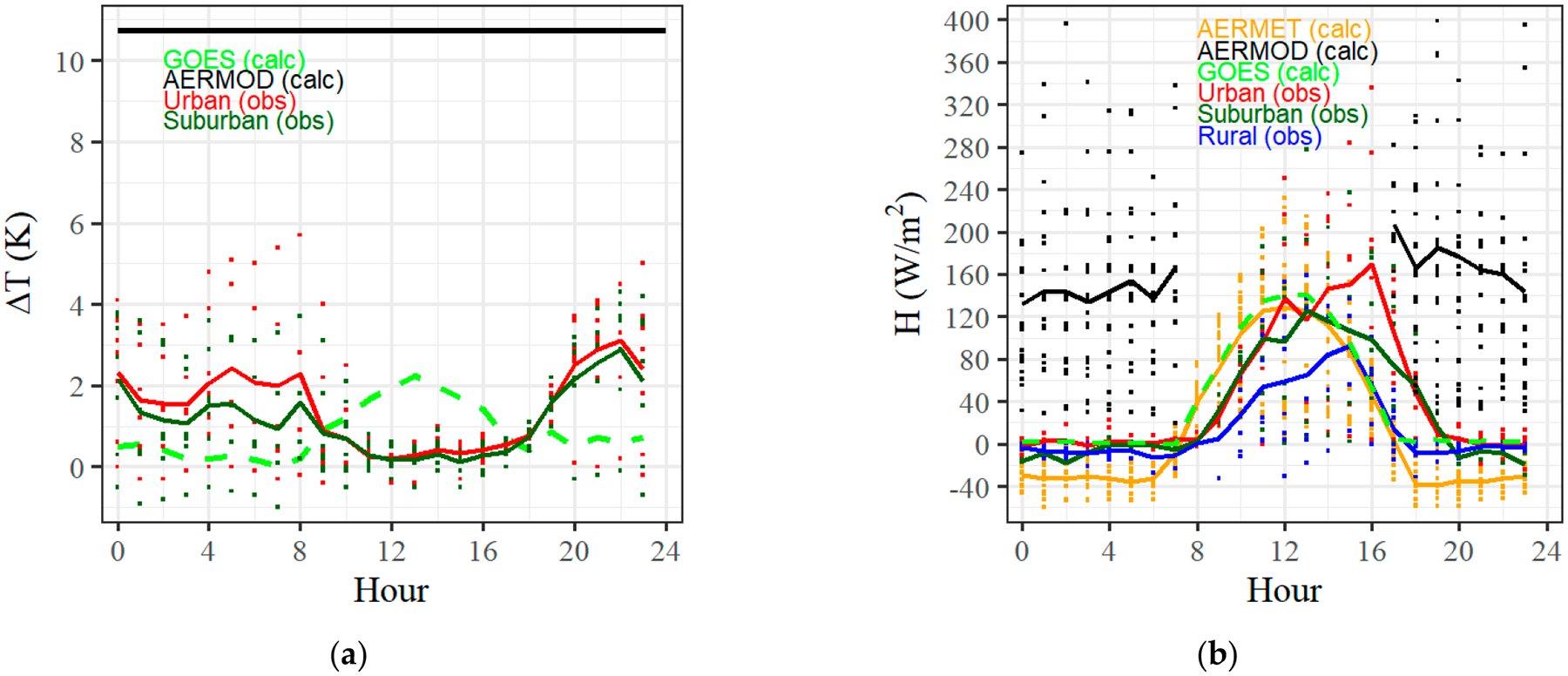
(**a**) ΔT measurements recorded from the Indianapolis field study. The urban and suburban results were referenced to the rural temperature. (**b**) Diurnal sensible heat flux observations and predictions from AERMET (rural option) and AERMOD (urban option). Lines are the average and points are instantaneous observations referenced to local time.

We now revisit the Indianapolis dataset, comparing the AERMOD urban option performance to the observations with and without our proposed heat flux formulation. Figure 7 compares observations from the kinematic heat flux sensors present in urban, suburban, and rural locations during the Indianapolis study to model predictions from AERMOD/AERMET and GOES-16 observations from 2021. The AERMOD case was run with a population input of 700,000 [40], whereas the AERMET values were from the meteorological surface file on SCRAM [42].

Figure 7a contrasts the predicted (calc) and observed (obs) ΔT values for the Indianapolis study. The predicted ΔT from AERMOD using Equation (7) is 10.7 K for all nocturnal hours. The measured urban and suburban air ΔT values peak at night with magnitudes < 29% of the AERMOD prediction. The GOES-16 land surface ΔT values are similar in magnitude to the urban and suburban air measurements but peak during the day, with minimum values at night. This temporal difference in air and land surface ΔT is typical for the main climate zones seen in CONUS other than arid climates (see Figure S2 in the Supplemental Material). For heat flux determination, one ideally needs both land surface and air temperature throughout the day, but routinely only one of the two is available.

Figure 7b illustrates these ΔT values applied directly as sensible heat flux with lines representing the hourly average for each method over the duration of the Indianapolis study. The outlier is the AERMOD predicted nocturnal heat flux, with a mean value of 157 W/m^2^ that is greater than the mean measured daytime heat flux during the experiment outside of the urban case at 16:00 local time. The rural predictions from AERMET show a near symmetric distribution around noon and a clearly stable nocturnal condition. The mean measured values from the experiment follow a similar diurnal trend while maintaining a hierarchy of rural, suburban, to urban heat flux magnitude throughout the day and night. The curves are not symmetric about noon, with a shift of ~2 h towards the later parts of the day for the suburban and urban heat flux curves with respect to the symmetric AERMET prediction.

The suggested improvements with the use of the GOES-16 LST data and Equation (9) improve the performance of the rural AERMET curve by (1) mitigating the strong negative heat flux at night to near neutral conditions and (2) modestly shifting the curve to later in the day, albeit not at the magnitude of the phase shift seen in the observations. Further edits are required to the net radiation model of AERMOD to properly diagnose this shift. However, the GOES-16-enabled curve best replicated the observed curves from the Indianapolis study.

## 5. Discussion

The AERMOD urban option was designed to alter the input meteorological conditions to improve the representation in the urban area. However, it does not reproduce the fundamental meteorological parameters observed in the urban area for the field study it was developed for. We now diagnose why the formulations were developed this way and how local scales of advection remain an issue with our directional ΔT approach.

### 5.1. Revisiting Model Evaluation with the Indianapolis Dataset

**Figure 8.**
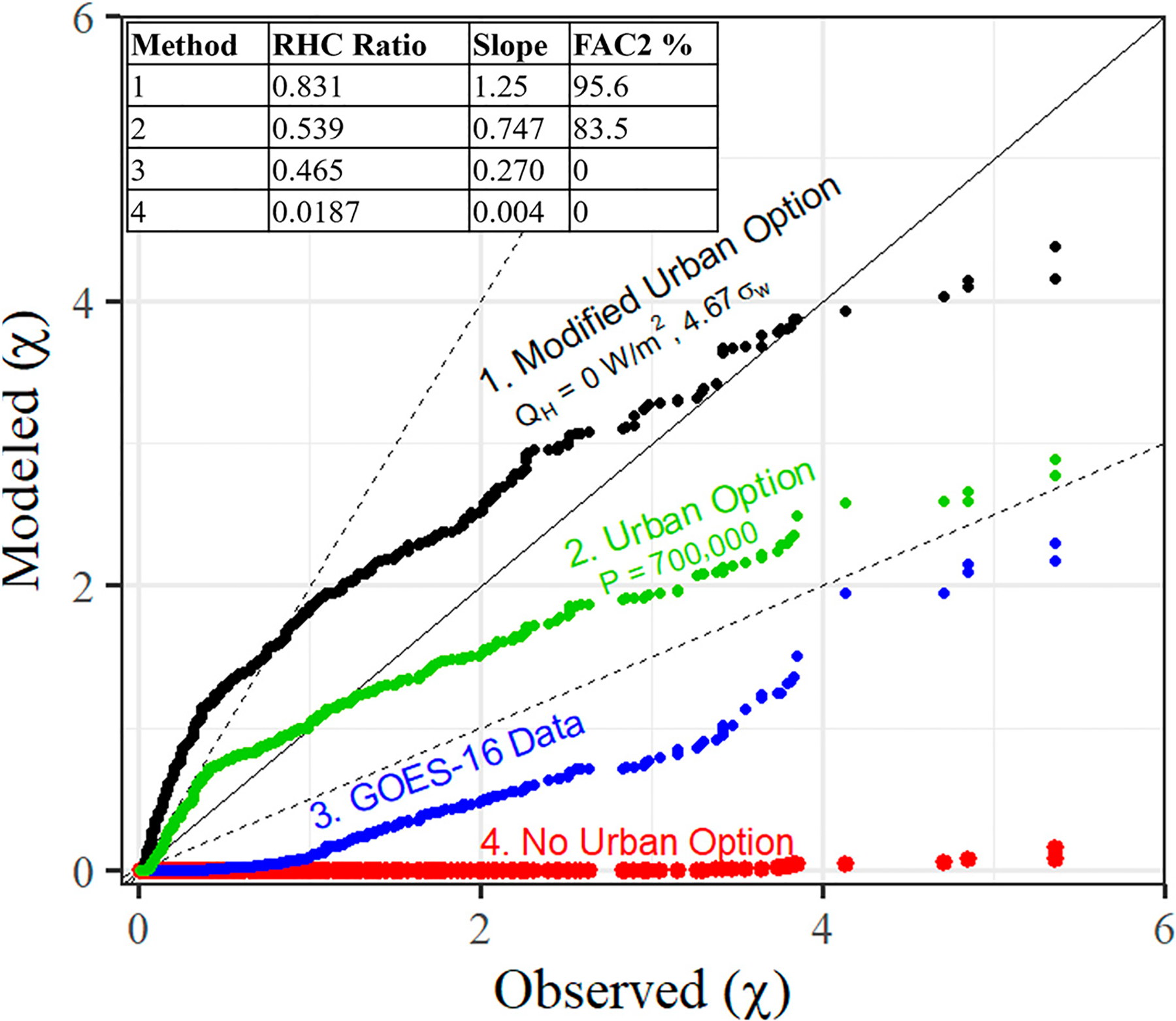
QQ plot of modeled versus observed concentrations with (1) a modified urban option with heat flux set to 0 W/m^2^ and increasing the vertical turbulence, (2) the urban option with a population of 700,000, (3) the urban option using the GOES-16 data to inform the heat flux as shown in Figure 7, and (4) without the urban option. Model performance for each method is included as a table embedded in the figure.

For regulatory models, the main interest is the peak concentrations as stated in the primary question of the AERMOD performance study by Perry et al. [40] of “how well does AERMOD predict the high-end, ground-level concentrations that are generally used to assess compliance with air quality regulations”? This is illustrated in the quantile–quantile (QQ) plot of Figure 8 for stable hours only for the AERMOD urban option, without the urban option, an urban option using the GOES-16 data from this work, and with a modified urban option we discuss here applied to the Indianapolis study. These data are ranked as observed and predicted arc maximums over the course of the experiment independent of the time or location of the observed or predicted value. Once again referencing Perry et al. [40], “for regulatory applications, a good model would produce a concentration distribution parallel to the slope of the measured distribution and produce high-end concentrations that are similar to that of the observations”. The robust highest concentration (RHC) statistic is used to quantify the high-end concentration predictions as

(10) $$\text{RHC}=\chi \{n\}+(\chi -\chi \{n\})\text{ln}\left(\frac{3n-1}{2}\right),$$

where χ{n} is the nth largest value, χ is the average of the highest n-1 values, and n is typically set to 26 [40]. The ratio of modeled (RHC_mod_) to observed (RHC_obs_) is used to characterize the model performance, where RHC_mod_/RHC_obs_ < 1 represents model underprediction. An ideal slope with respect to the QQ plot of Figure 8 is 1, where a slope < 1 demonstrates general model underprediction over the entire distribution. As a final means of general model performance, the percentage of predicted sorted concentrations within a factor of two (FAC2) of the sorted observations are also tabulated in the table within Figure 8. The no urban option features a slope of 0.004, RHC_mod_/RHC_obs_ = 0.0187, with FAC = 0%, validating the need to develop an urban option. The existing AERMOD urban option, using an input population of 700,000 representative of the year during the field study, improves model performance significantly for all parameters, with a best fit slope of 0.747, RHC_mod_/RHC_obs_ = 0.539, and FAC = 83.5%.

However, as shown in Section 4.2, this apparent success of utilizing the urban option is accomplished with unphysical inputs for the sensible heat flux. An independent analysis of the Indianapolis Study by Hanna and Chang [14,43] using onsite meteorology showed a negligible convective velocity scale at night (see Figure 18 in Hanna and Chang [14]) and the presence of a low-level jet (see Figure 19 in Hanna et al. [14]). They suggest increasing the mechanical vertical turbulence at night by a factor 4.67× (see Equations (19) and (20) in Hanna et al., where factor $a_{w}$ is increased from 0.15 to 0.70 at night) “to account for the nocturnal jet and for the turbulence that is present aloft during cloudy conditions” [43]. Comparing these two approaches reveals that the urban option in AERMOD is modifying the incorrect physical variable by adding convective turbulence to account for a low-level jet and not an urban heat island.

To further illustrate this point, we mimic the work of Hanna and Chang by setting the heat flux to 0 W/m^2^ at night and increasing the vertical mechanical turbulence by 4.67× in AERMOD. This produces the modified urban option curve in Figure 8 with a slope of 1.25, RHC_mod_/RHC_obs_ = 0.831, and FAC2 = 95.6%. The results are more conservative, and each performance parameter is improved while changing the correct physical variable. This suggests that updates are required to AERMOD’s handling of mechanical turbulence in urban areas, especially in the presence of a low-level jet. As a final point of comparison, using the GOES-16 data for Indianapolis, which produces the realistic heat flux curves in Figure 7, offers improved model performance from the baseline, though this update underpredicts when compared to both the current urban option as well as the measured concentrations. This further suggests that the incorrect parameter (sensible heat flux) was adjusted in AERMOD to improve model performance in urban areas. Mechanical turbulence is the cause of increased concentrations in the Indianapolis database, not convective turbulence. Therefore, future work will entail updates to the urban mechanical turbulence formulation and detailed comparisons to computational fluid dynamics modeling efforts.

### 5.2. Scales of Advection

While the use of ΔT observations within Equation (9) will produce sensible heat flux curves that are more representative than the existing population-based method (see center column of Figure 6) and offer directional, seasonal, and diurnal city-specific observations, several questions remain. We continue to group urban areas into similar pixels, but is the local space/time error of assuming one value of sensible heat flux for an entire city every hour acceptable for these reduced order Gaussian dispersion algorithms? There are clearly spatial variances within a city, leading to local scales of advection. How does this differ from a city-averaged approach? These topics are discussed here.

**Figure 9.**
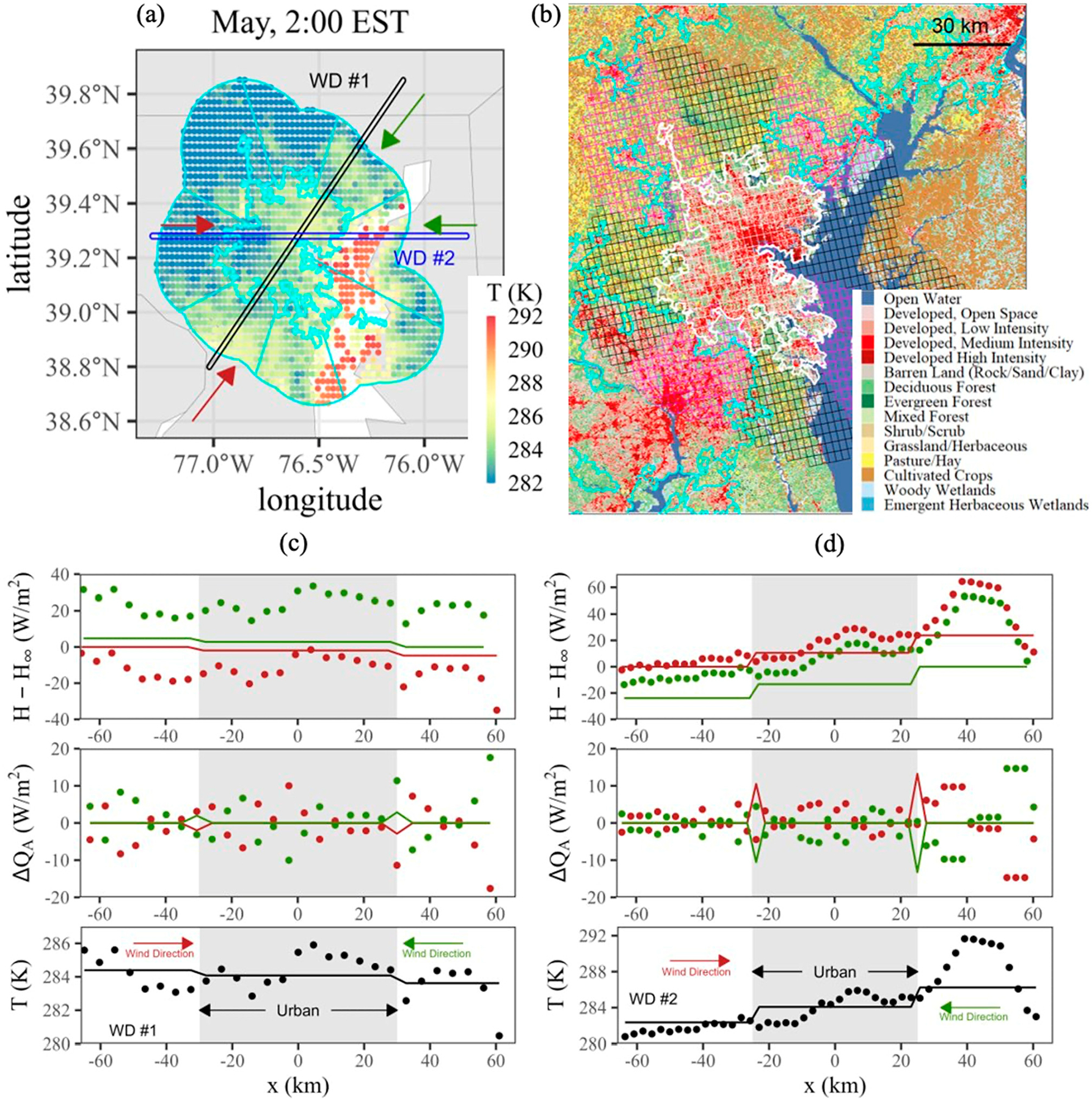
Case study of advective scales in Baltimore, Maryland at 2:00 a.m. EST in May 2021. (**a**) Monthly averaged LST and SST for the Baltimore urban area and surrounding sectors with highlights of two different wind paths through the city center (WD #1 and WD #2) with wind directions marked as red or green. (**b**) Corresponding NLCD data for Baltimore. (**c**) Analysis of WD #1 from (**a**): Surface temperature (**bottom**), corresponding horizontal advection using Equation (9) for each wind direction (**middle**), and cumulative heat flux with respect to a reference value located at the boundary (**top**) at the GOES resolution (points) and sector-averaged resolution (lines). (**d**) Similar to (**c**) for WD #2 from (**a**).

This study provides a city-scale representation of the advection of sensible heat flux and neglects, by means of spatial averaging, local scale advection. To examine these effects, a small case study was considered in Figure 9 for Baltimore at 2:00 a.m. EST in May 2021 for two different wind paths shown in Figure 9a. These highlight both directional effects towards nearby cities (WD #1, Figure 9c as illustrated in Figure 9a) and the local influence of bodies of water (WD #2, Figure 9d, as illustrated in Figure 9a), as seen with the NLCD data for the area in Figure 9b.

Both examples shown in Figure 9 oppose the illustration of an ideal urban environment, like that shown previously in Figure 1, where similar land cover is observed both upwind and downwind of the urban area. For WD #1 in Figure 9 along the southwest-northeast axis of the city, the surface temperature is generally decreasing (or increasing) from the south to the north (north to the south) due to the influence of Washington, DC to the southwest, as seen previously in Figure 5. Using the average sector approach suggested for use with AERMOD, and as seen as lines in Figure 9c, produces modest step changes in temperature and sensible heat advection that result in small changes to the heat flux with respect to a reference value at the border of the domain ($H_{\infty }$) that mimics the spatial temperature profile. More dynamic changes are observed using the full resolution of the GOES LST data represented by the points in Figure 9c by explicitly applying Equation (9). Refined spatial scales clearly deviate from the sector averaged approach both in the surrounding areas and within the US Census-defined urban area.

A similar situation is observed for WD #2 in Figure 9d where the water temperature of the Chesapeake Bay to the east is warmer than the land at night, leading to a large positive advective component with flow from the west with a likewise negative advective component with flow from the east. In comparison to WD #1, these spatial curves are less chaotic, likely representing the relative simplicity of the surrounding ground cover (water to the east, rural land to the west) in comparison to the urban areas surrounding Baltimore to the southwest and northeast. The average sector approach, while again limited in magnitude, provides the general directional trend present in the raw data.

To accurately account for these local effects would require a more complex, pre-processor-like tool to best characterize the local urban area near the dispersion source to be modeled. This could mimic the directional fetch land cover analysis performed to estimate the albedo, surface roughness, and Bowen ratio already utilized by AERMET [28]. The averaged approach presented here offers a city-scale compromise to improve the existing diurnal heat flux profiles present in AERMOD.

## 6. Conclusions

Calculating the urban sensible heat flux is a complicated task given the complexities in the air and surface temperature differences in urban areas with respect to their surroundings. We focused on comparisons of the urban parameterization in AERMOD, based on a single population input, to recommendations from the literature and an advective approach based on surface ΔT observations from GOES-16 ABI products. We developed a clear sky only, monthly averaged, hourly land and sea surface ΔT database to help estimate city-specific differences in diurnal sensible heat flux profiles for each US Census-defined urban area in CONUS from 2010. Eight sectors surrounding each city were selected to account for changes in fetch of the boundary layer entering the city. The national land cover database from 2019 was used to characterize all urban areas and each surrounding sector.

Comparisons of AERMOD predictions to GOES-16 observations were highlighted for the four cities of Cleveland, Amarillo, Atlanta, and Baltimore for both ΔT and sensible heat flux. The AERMOD formulation produces a constant ΔT at night for a given population, resulting in predicted values 794%, 416%, 1048%, and 758% higher than the GOES-16 observations for Cleveland, Amarillo, Atlanta, and Baltimore, respectively. We then investigated (1) implementing the ΔT observations to estimate city-specific corrections to the city-generic heat balance model in AERMOD and (2) setting the storage heat flux at night to the net radiation, as suggested in the literature. This enables the advection corrections to set the stability class of the urban area at night, resulting in convective conditions 53%, 92%, 62%, and 55% of the time with mean nighttime sensible heat flux of −0.8 W/m^2^, 8.6 W/m^2^, 3.0 W/m^2^, and 3.1 W/m^2^ in Cleveland, Amarillo, Atlanta, and Baltimore, respectively, which are consistent with observations in the literature. These are juxtaposed with the existing AERMOD urban option, which produced inflated values of sensible heat flux with predictions of 120.8 W/m^2^, 127.1 W/m^2^, 115.0 W/m^2^, and 126 W/m^2^, respectively, while forcing a stable classification.

Comparison of AERMOD with the Indianapolis Power Plant Plume Study was revisited to understand the origin of the existing urban formulations. Observed average air ΔT values were <29% of the AERMOD predicted value of 10.7 K, translating to a mean nocturnal sensible heat flux prediction of 157 W/m^2^ from AERMOD. This is well above the near zero value measured for suburban and urban sites and is above daytime observed values. Even with erroneous heat flux predictions, AERMOD performs well when compared to sorted nocturnal concentrations in a QQ plot, as the peak values are of interest for regulatory purposes. An independent analysis of the Indianapolis study showed this increase in concentration is due to a low-level jet increasing vertical turbulence and not by convective means. This suggests that the “convective like boundary layer” approach in the AERMOD urban option is an inaccurate portrayal of the Indianapolis study.

The corrections suggested in this work will produce improved sensible heat flux curves in the urban option of AERMOD. However, they remain as reduced order city-scale parameterizations dependent on clear sky datasets and require future validation from higher fidelity convective simulations or field studies. Adoption by AERMOD would make the urban option meteorology more realistic but will require additional enhancement of the mechanical turbulence to best represent increased surface roughness in urban areas. Future work will include incorporating wind tunnel measurements in urban areas and computational fluid dynamics simulations to improve the mechanical turbulence parameterization in the AERMOD urban option.

## Supplementary Material

Supplement1

Supplementary Materials: The following supporting information can be downloaded at: https://www.mdpi.com/article/10.3390/atmos16121342/s1, Figure S1: Diurnal, monthly averaged, clear sky only urban–surrounding surface temperature differences for (a) Cleveland, (b) Amarillo, (c) Atlanta, and (d) Baltimore. Each subplot represents the source wind direction for classification of the surroundings. Figure S2: Average diurnal ΔT profiles for each major climate zone shown in Figure 3b in the main paper for each month in 2021. Hours on the x-axis are presented as the local for each city. Note that climate zone A curves were divided by 5 to fit on the same plot. Figure S3: Monthly averaged diurnal ΔT profiles sorted by surrounding land cover class. Note that barren land and water curves are divided by 3 to fit on the same plot. Figure S4: Coastal city directional ground cover for (a) Los Angeles, (b) Boston, and (c) Tampa Bay. The GOES-16 LST/SST grid is added to the NCLD plot on the bottom row to illustrate the spatial scales of the temperature measurement with respect to the land cover data. Figure S5: Example ΔT, sensible heat flux, and directional nocturnal stability for three coastal cities.

## Figures and Tables

**Figure A1.**
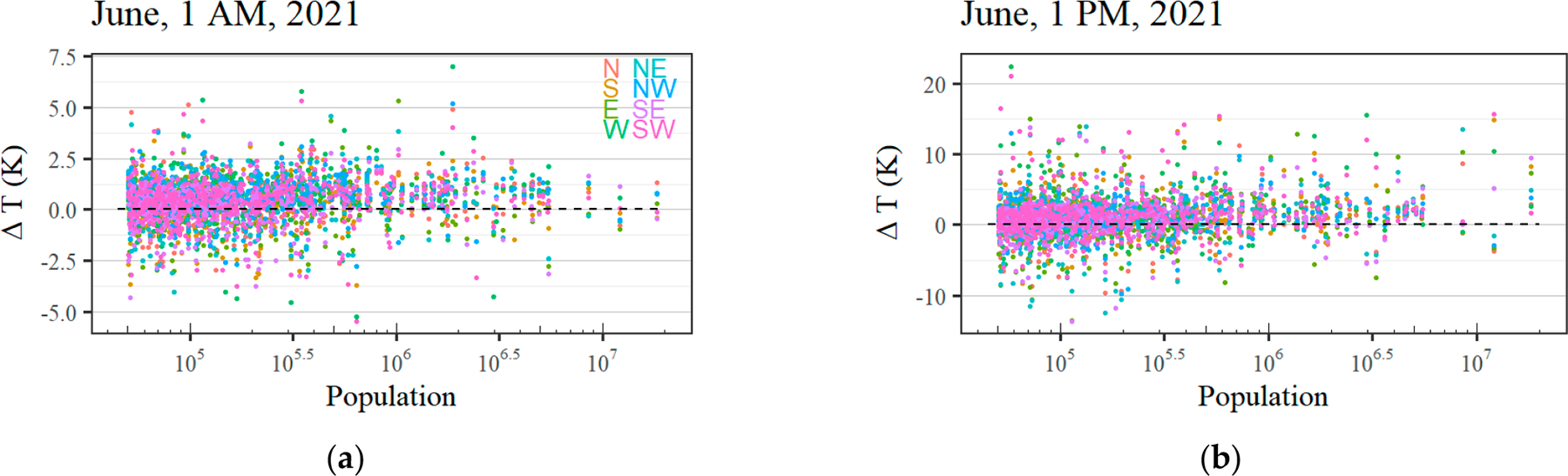
(**a**) Observed mean ΔT for all wind directions for each city at 1 a.m. local time in June against the population. (**b**) Similar, only for daytime at 1 p.m. local time. Note the difference in scale on the y-axis for night- and day-time.

## Data Availability

The data that support the findings of this study will be openly available on data.gov upon acceptance to the journal.

## Appendix A. On the Issue of a Constant ΔT

The current approach in AERMOD of air ΔT=constant at night and ΔT=0 during the day is a simplification and ignores observed land surface values presented in this work. This prevents properly accounting for advective contributions to sensible heat flux in horizontally inhomogeneous (urban–surrounding) areas, as discussed in the main section of the paper. Figure A1 highlights the dependence of population on the range of ΔT values seen for each city when considering all wind directions both at night and during the day. For all 480 urban areas, 63.3% and 63.8% of cities span both negative and positive ΔT for the 1 a.m. and 1 p.m. cases, respectfully, illustrating the difficulty of parameterization with a single population value.
