# Supplementary material for: Revisiting the Convective Like Boundary Layer Assumption in the Urban Option of AERMOD
Source: Atmosphere (Basel). Author manuscript; Available in PMC 2026 Feb 25. (PMC12931099; doi:10.3390/atmos16121342)
Supplement: Supplement1 [file NIHMS2137399-supplement-Supplement1.pdf]

Supplementary Materials:

Jonathan Retter [1,2], Robert Christopher Owen [2,*], Annamarie Leske [3], Michelle Snyder [4], Rhett Sargent [5] and David Heist [2]

[1] Oak Ridge Institute for Science and Education Research, Research Triangle Park, NC 27711, USA; jretter92@gmail.com
[2] U.S. EPA Office of Research and Development, Center for Environmental Measurement and Modeling, Research Triangle Park, NC 27711, USA; heist.david@epa.gov
[3] Johns Hopkins Applied Physics Labs, Laurel, MD 20723, USA
[4] WSP Global Inc., Durham, NC 27703, USA; michelle.snyder@wsp.com
[5] Department of Statistics, Virginia Tech University, Blacksburg, VA 24061, USA
* Correspondence: owen.chris@epa.gov

1. Surface Urban Heat Island Results

The main paper focuses on surface UHI ΔT observations applied as advection corrections in the urban energy balance model of AERMOD. This supplemental section highlights the ΔT observations themselves, where the sector-based averaging procedures using LST and SST, as described in Section 3 of the main paper, are performed for 480 urban areas in CONUS. Individual temperature differences for each city (Section 1.1) are tabulated in input files for future use and comparisons are made for climate zone (Section 1.2) and ground cover dependence (Section 1.3). Finally, the application of these ΔT observations for sensible heat flux parameterization is demonstrated in four coastal cities in Section 1.4 to highlight the effect of sea surface temperature.

1.1. Individual City Examples

Figure S1 illustrates the monthly averaged, diurnal, and directional urban–surrounding LST differences corresponding to the four cities highlighted in the paper of Cleveland, Amarillo, Atlanta, and Baltimore. These are all direct observations based on the methods presented in Section 3 of the paper and are depicted here to illustrate the difficultly in accurately modeling this city-specific, seasonally varying change in temperature with a single population input. The variance in observed LST values with population is examined further in Appendix A of the main paper. The results for each city are included as text files on data.gov for reader convenience (see data availability statement).

Strong diurnal trends for wind directions from the northeast through the west are present in Figure S1a for Cleveland, a snow climate city, where flow into the city originates from Lake Erie. ΔT values are positive throughout the day, peaking near 14:00 local time, with maximum values ($\Delta T > 10\ K$) occuring during the spring months of March–May and minimum values during the winter in November–January ($\Delta \mathrm{T} < 2\ K$). Nighttime hours for these water-sourced wind directions result in $\Delta T < 0\ K$, as the higher thermal capacity of water with respect to land prevents the water surface temperature from evolving as quickly as the land cover in the city. Winds from the south produce a slight diurnal pattern, where the ΔT decreases into the day due to the elevated LST from Akron, Ohio. All other wind directions have slight seasonal variations with near constant diurnal patterns, with peak values occurring in the late summer or fall months ($\Delta T \sim 2\ K$) and nearly no surface ΔT during winter months.

Amarillo, Texas acts as a representative for the arid climate urban areas in Figure S1b and illustrates a peak ΔT at night or even early morning for most surrounding sectors and months. Routine urban cool islands ($\Delta T < 0\ K$) are observed in the middle of the day, particularly from the west and southwest for summer months. Directly opposing this for the same season, sectors from the east of the city feature a positive ΔT during the day that exceed nighttime values for summer months, although there is no obvious reason in the corresponding ground cover from Figure 4 in the main paper to describe this behavior in comparison to other sectors.

Figure S1c depicts Atlanta, Georgia, the representative from the humid temperate climate zone. No significant trends are observed, as all sectors and seasons have small peaks during the day near ~2 K and decay to near zero at night. This is a rare example where a population number might be effective at describing the heat island characteristics of the city, as the curves are consistent across the seasons and directions.

Finally, Figure S1d represents Baltimore, Maryland, a city selected specifically due to the complicated nature of its surrounding geography. Flow from the southwest is altered by the larger city of Washington, DC, thereby causing an urban cool island in Baltimore for nearly all hours for each month. East and southeast sectors include large portions of the Chesapeake Bay which, like Cleveland, remain relatively stable in temperature, leading to peak ΔT values during the day for spring and summer months with negative values at night. From the northeast and west, the boundary layer traverses over smaller urban areas, albeit not the size of Washington, DC, leading to a positive diurnal ΔT, but less than a true rural reference case such as flow from the north.

1.2. Dependence on Climate Zone

To generalize guidance on surface ΔT values, Figure S2 represents monthly averaged diurnal profiles for each major climate zone in CONUS shown previously in Figure 3b. The mean ΔT over all wind directions was used for each city. Values for climate zone A (equatorial) are divided by 5 to reach similar magnitudes for plotting purposes, as only two CONUS urban areas with very similar environments exist in climate zone A (Bonita Springs and Miami, both in Florida). These equatorial urban areas feature urban cool islands at night, with $\Delta T < 0$ for all months, due to the prominence of nearby ocean water. Values peak near 14:00 local time and decay slowly into the evening, demonstrating how urban surfaces remain hotter longer into the day.

Climate zone Cs (summer dry temperate, west coast of CONUS) coincides with the equatorial trends during the winter months ($\Delta T < 0$ at night, high bias in evening hours) likely due to similar effects of nearby ocean water on the opposite coast. However, its diurnal profile shifts through spring to a unique shape in summer, featuring a gradual decay from a peak at 14:00 local time until becoming negative in the morning hours before a steep rise during the day. This is likely an artifact from averaging over both coastal and inland cities within the same climate zone, where coastal ΔT values overcome small inland values during the winter months, resulting in $\Delta T < 0$ at night. For summer nights, inland values match the coastal values, albeit with a different temporal profile resulting in, on average, a morning cool island.

Climate zones Cf (humid temperate) and D (snow) represent the south and north, respectively, of the east coast of the CONUS. Their average diurnal ΔT profiles all peak during the day with small values at night, representing the traditional view of *surface* ΔT trends. Conversely, climate zone B (arid) is more representative of how *air* ΔT values are described in the literature for spring through fall months, where values peak at night with minimum values during the day [1]. This arid diurnal trend was also observed by Chakraborty et al. with polar-orbiting satellite imaging offering discrete observations during the day- and nighttime [2]. Zhao et al. [3] attribute this to increased convective efficiency in arid urban areas, as the aerodynamic roughness of cities is greater than their surroundings, leading to more efficient removal of heat and thus, lower or negative ΔT values during the day.

1.3. Dependence on Land Cover

An alternative method to generalize representative urban ΔT values is by land cover class from the 2019 NLCD shown previously in Section 3.1.2. As opposed to climate zone divisions, this method allows all wind direction sectors for each urban area to be analyzed separately. Figure S3 illustrates these diurnal profiles by taking the most common land cover type in each wind direction around every city and averaging it with all other occurrences of the same land cover for the same hour and month. Given the 30 km spatial buffer

considered for this work, nearly all surrounding sectors are a mix of land cover classes; therefore, this approach is a simplifying estimate. Open water and barren land had significantly high or low values, respectively, representing the largest influence on directional ΔT and were divided by 3 for ease of visualization.

The general trend for most of the land cover types is a Gaussian-like curve with tails in the late evening/early morning, and a peak in the early–mid-afternoon, juxtaposed by barren land with an inverse profile and developed land with a constant, slightly negative ΔT profile for all months throughout the year. The barren land profile parallels that of climate zone B from Figure S2 (arid). The importance of seasonal variation is illustrated with cultivated crop, grassland, and shrub land cover profiles, where similar behavior to barren land is seen in late spring through the summer with negative values during the morning and peak values at night. Both woody and emergent herbaceous wetlands, pastures, and cultivated crop lands cover classes occasionally feature local minima before and after the peak value in the afternoon. Finally, the open water profile reliably changes sign from negative at night to positive during the day throughout the year, as again nearby water offers a stabilizing temperature to the comparatively dynamic urban area land temperature.

1.4. Coastal City Examples

Incorporating SST observations enables the directional analysis of coastal cities. Los Angeles, Boston, and Tampa Bay are viewed here, applying the same procedure as discussed throughout this work. Figure S4 details the directional land cover classes for each city and corresponding NLCD maps overlaid with the GOES-16 surface temperature resolution grid. Directional sectors are visualized by alternating colors, beginning with north in black. The US Census defined urban area is highlighted in white, whereas surrounding urban areas are highlighted in blue. Tampa Bay highlights an unusual city shape where this crude directional approach leads to issues with the water cover.

Figure S5 mimics Figure 6 in the main section of the paper and shows the monthly averaged ΔT (left column), sensible heat flux (middle), and nocturnal stability class based on wind direction (right column) for these three coastal cities. The existing AERMOD values are shown in thin, faded lines against the results of this work in thick lines. Dashed heat flux curves are the rural default outputs from AERMET. All monthly averaged values are weighted by the wind direction, producing the expected ΔT peak during daytime hours as these cities are not in arid climates where the reverse trend is expected. The nocturnal stability is directly connected to the relevant surrounding sector, as all water-based surroundings produce more stable conditions whereas surrounding land leads to convective conditions. Note that the west direction for Los Angeles includes both water and land, therefore, leading to a mixed stability classification throughout the year.

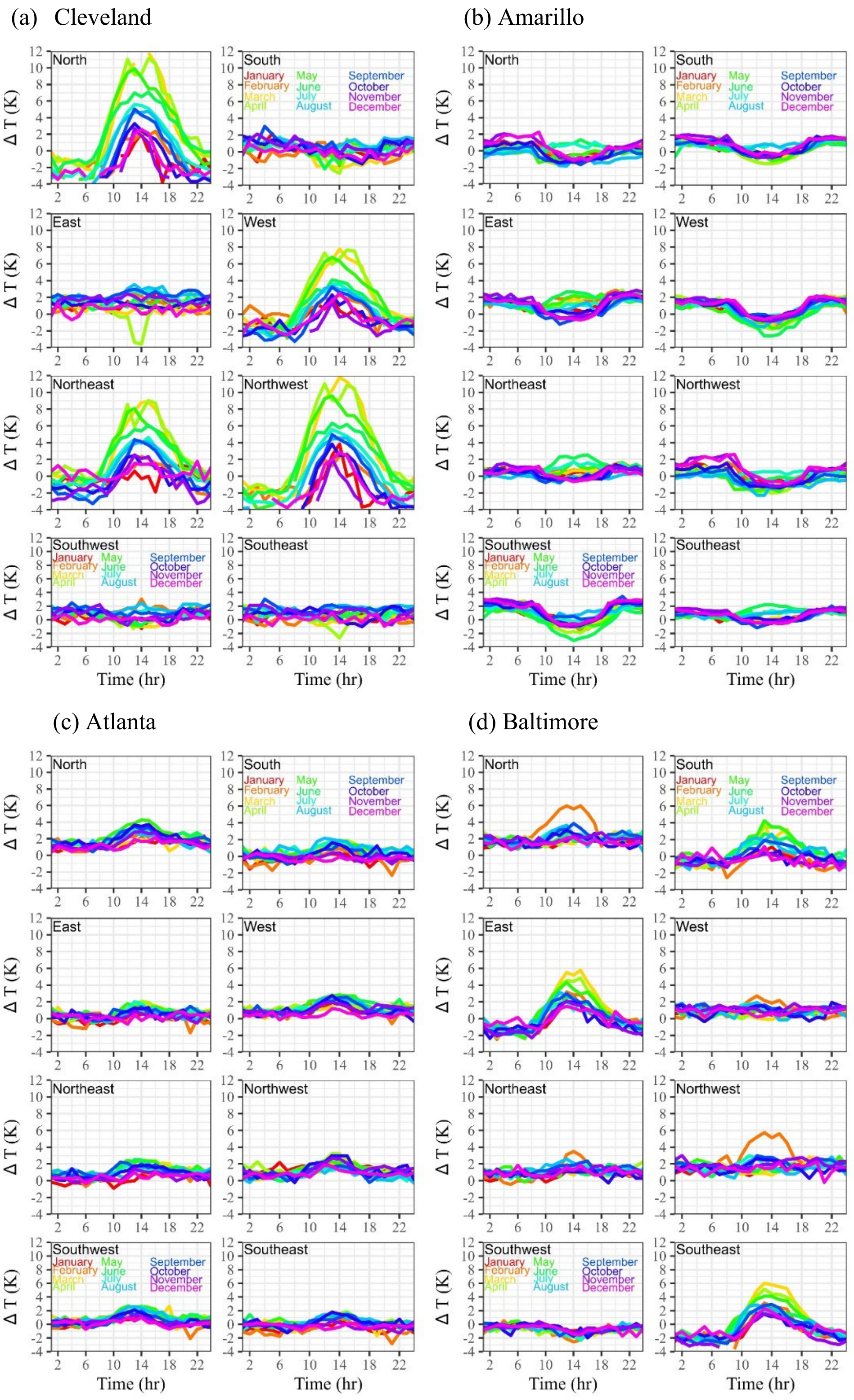

Figure S1. Diurnal, monthly averaged, clear sky only urban–surrounding surface temperature differences for (a) Cleveland, (b) Amarillo, (c) Atlanta, and (d) Baltimore. Each subplot represents the source wind direction for classification of the surroundings. .

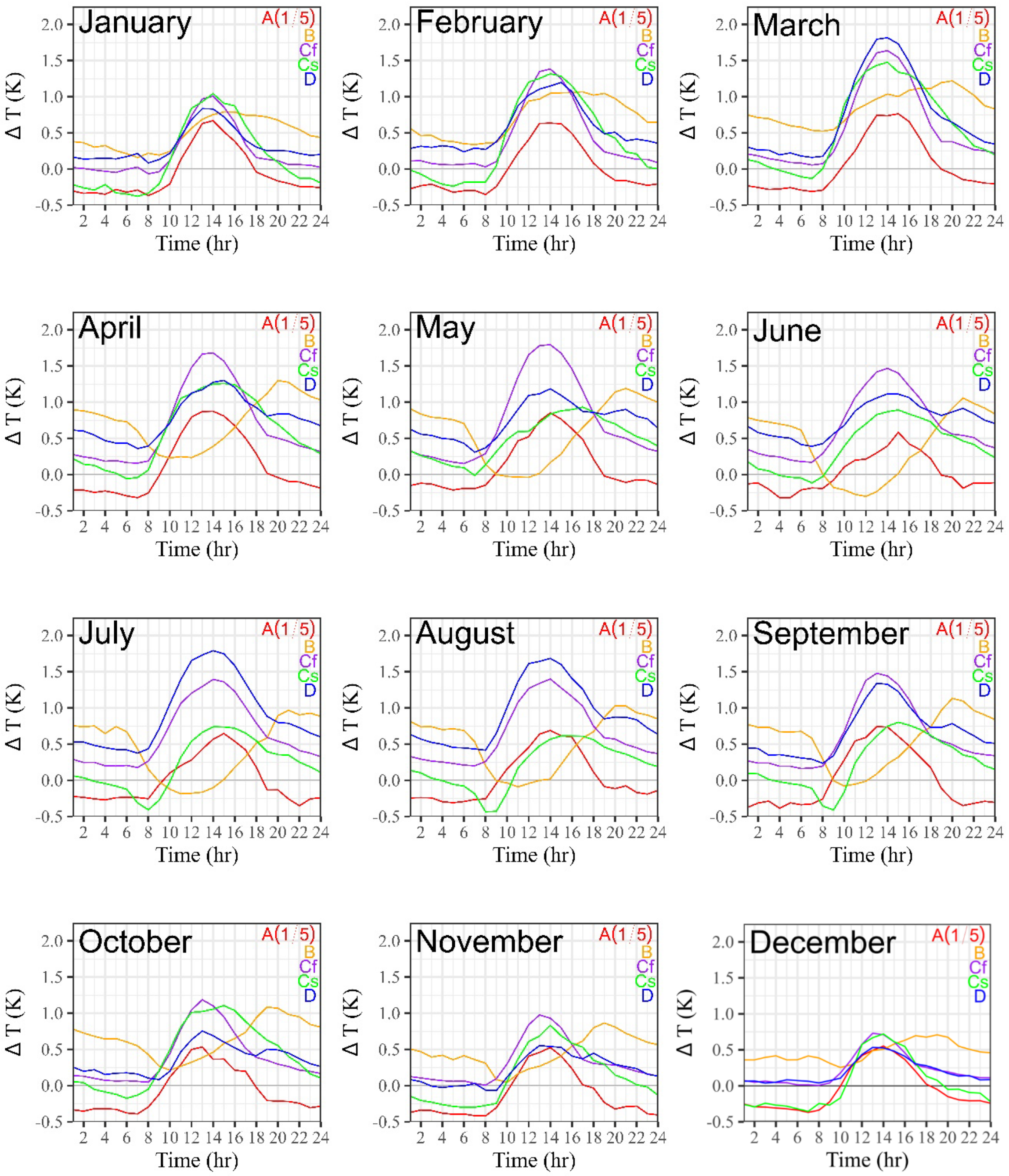

Figure S2. Average diurnal ΔT profiles for each major climate zone shown in Figure 3bb in the main paper for each month in 2021. Hours on the x-axis are presented as the local for each city. Note that climate zone A curves were divided by 5 to fit on the same plot.

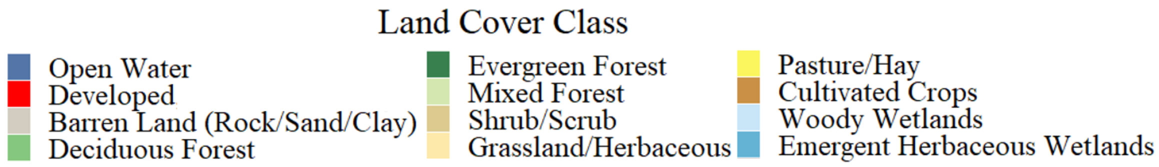

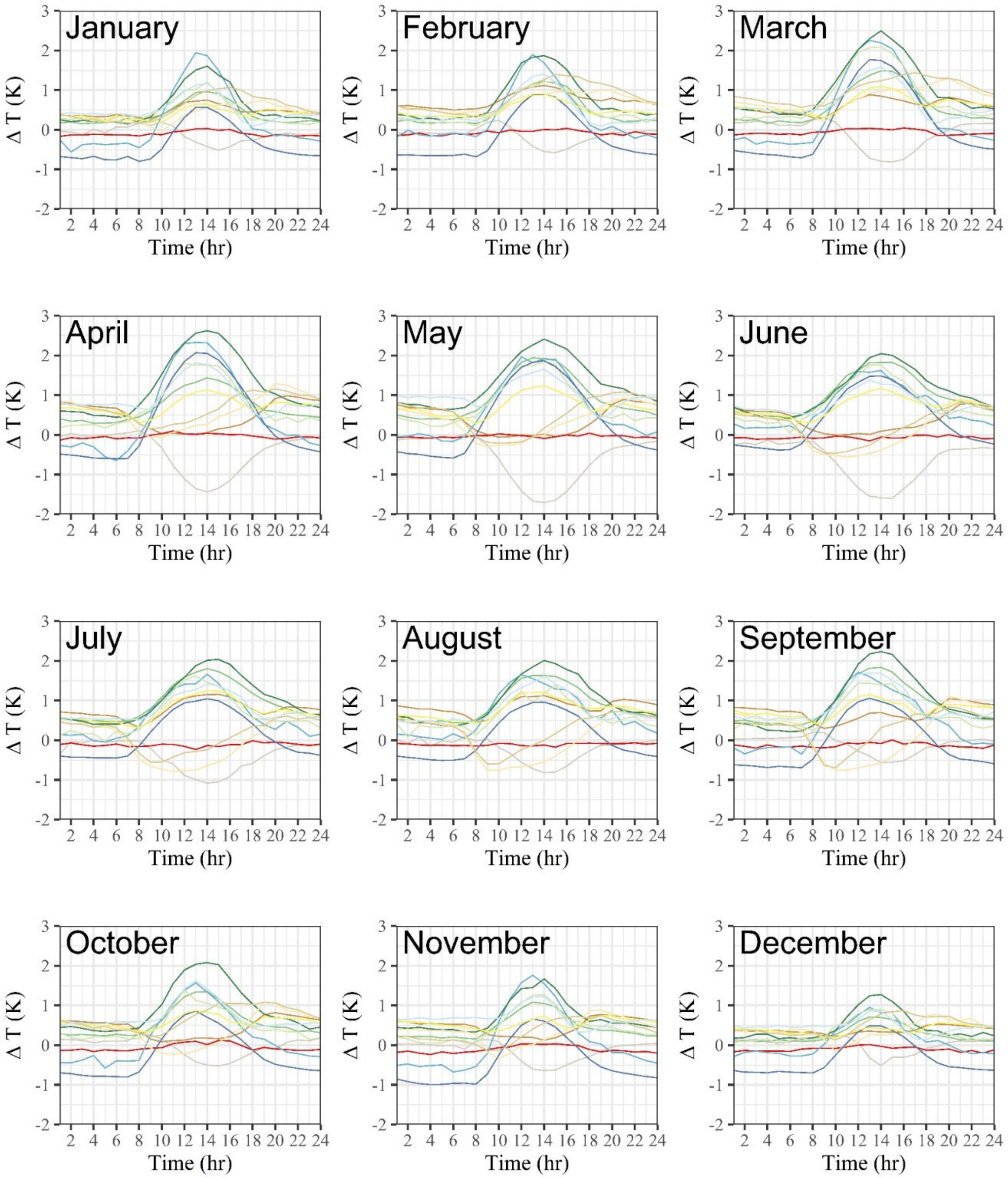

Figure S3. Monthly averaged diurnal ΔT profiles sorted by surrounding land cover class. Note that barren land and water curves are divided by 3 to fit on the same plot.

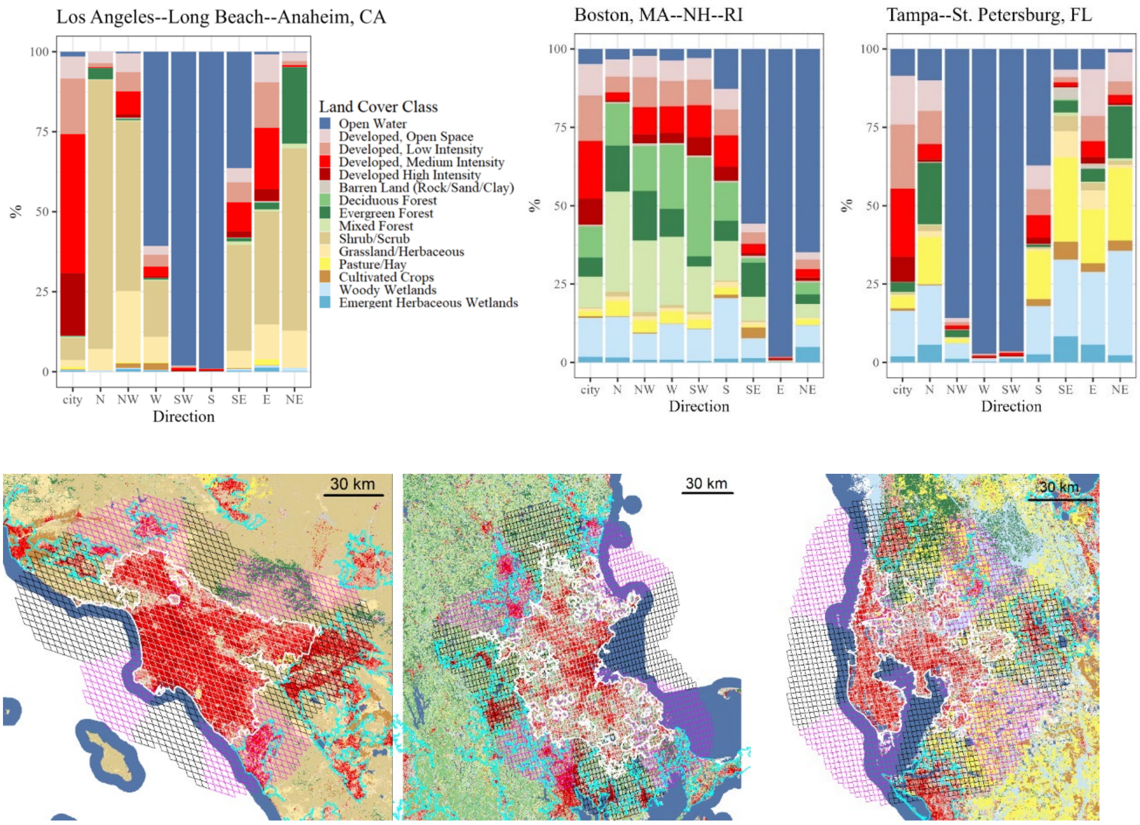

Figure S4. Coastal city directional ground cover for (a) Los Angeles, (b) Boston, and (c) Tampa Bay. The GOES-16 LST/SST grid is added to the NCLD plot on the bottom row to illustrate the spatial scales of the temperature measurement with respect to the land cover data.

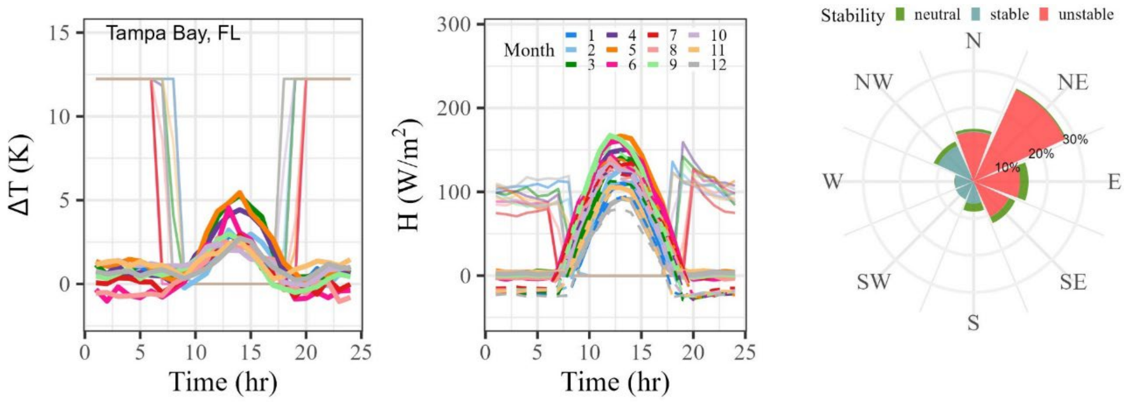

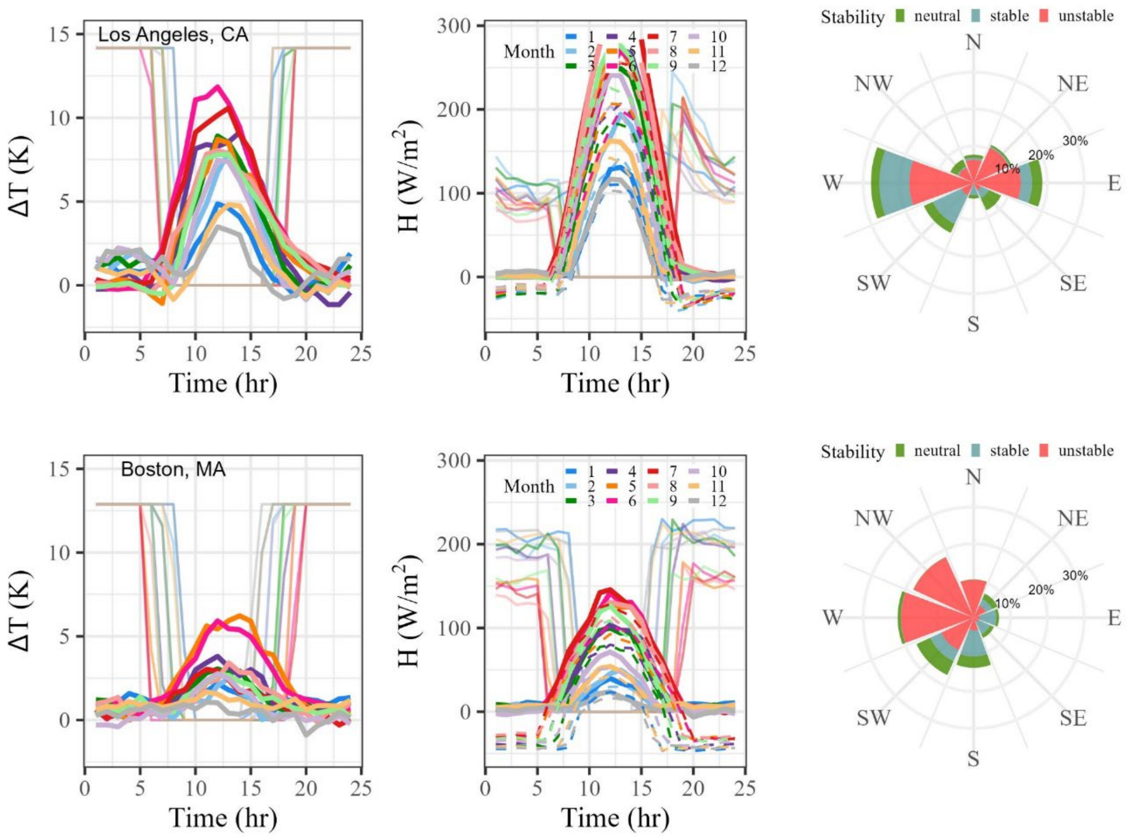

Figure S5. Example ΔT, sensible heat flux, and directional nocturnal stability for three coastal cities.

Reference

1. Lazzarini, M., P.R. Marpu, and H. Ghedira, Temperature-land cover interactions: The inversion of urban heat island phe-nomenon in desert city areas. Remote Sensing of Environment, 2013. 130: p. 136-152.
2. Chakraborty, T. and X. Lee, A simplified urban-extent algorithm to characterize surface urban heat islands on a global scale and examine vegetation control on their spatiotemporal variability. International Journal of Applied Earth Observation and Geoin-formation, 2019. 74: p. 269-280.
3. Zhao, L.; Lee, X.; Smith, R.B.; Oleson, K. Strong contributions of local background climate to urban heat islands. *Nature* **2014**, *511*, 216–219.

Disclaimer/Publisher's Note: The statements, opinions and data contained in all publications are solely those of the individual author(s) and contributor(s) and not of MDPI and/or the editor(s). MDPI and/or the editor(s) disclaim responsibility for any injury to people or property resulting from any ideas, methods, instructions or products referred to in the content.
